# Exploring novel NH-form resorcinol-based schiff base and its metal complexes: Synthesis, characterization, cytotoxic activity, molecular docking and ADME studies

**DOI:** 10.1016/j.heliyon.2024.e37385

**Published:** 2024-09-07

**Authors:** Elham S. Aazam, Maryam Majrashi, Mostafa A. Hussien

**Affiliations:** a Chemistry Department, Faculty of Science, King Abdulaziz University, Jeddah, 21589, B.O. Box 80203, Saudi Arabia; b Department of Chemistry, Faculty of Science, Port Said University, Port Said, 42521, Egypt

**Keywords:** MCF-7, X-ray crystallography, NH schiff base, Metal complexes, 5-FU, MTT assay, Antiproliferative, Resorcinol, Molecular docking, ADME

## Abstract

The research investigates the cytotoxic effects of the stable NH-form of a resorcinol-based Schiff base (HL) and its metal complexes (Zn(II), Cd(II), Cu(II), Ni(II)) on MCF-7 breast cancer cells. The structural characterization was conducted utilizing diverse analytical techniques, including mass spectrometry, elemental analysis, molar conductance, magnetic moment, UV–Vis, IR and ESR. The crystalline state analysis of HL through X-ray crystallography disclosed a hybrid structure comprising two canonical forms, specifically the quinoid and zwitterion, that contribute to resonance and diverse interactions, resulting in the development of a three-dimensional form. NMR, IR and ESR analyses showed that the HL was bidentate, using the oxygen of the hydroxyl and the nitrogen atom of azomethine, bonded to the metal center during complexation. The study explored the cytotoxic effects of HL and the various metal complexes on MCF-7 human breast cancer cells. All complexes display significant cytotoxicity (IC_50_ < 38.37 μM). The activity of the complexes was greater than that of the free ligand, with the Cu(II) complex followed by Zn(II) demonstrated superior cytotoxicity compared to Cd(II), and Ni(II) complexes. Notably, the Cu(II) and Zn(II) complex exhibited approximately 13.2 and 12.9 times greater cytotoxicity than the 5-F Uracil (5-FU) cancer drug. An MTT assay corroborated the antiproliferative activity. The molecular docking study has been performed for all compounds with the aromatase cytochrome P450 receptor protein associated with breast cancer (PDB code = 3eqm). ADME drug likeness model has been done.

## 1 Introduction

In recent years, the surge in cancer cases has prompted the exploration of novel treatments. Cancer, a leading global cause of death, necessitates the development of innovative molecules to address multidrug resistance. Small molecules, with the potential as anti-cancer, already exist, but new molecules are needed to overcome resistance. Metallo-drugs, particularly Schiff-based ones, have shown success in antiproliferative chemotherapy and hold promise for more efficient treatment of multi-resistant infections [1].

Schiff bases are extensively researched due to their diverse characteristics, including thermochromic and photochromic behaviors [2], as well as tautomeric equilibria involving proton shifts [3], pharmacological effects [4], and analytical applications [5]. Their capacity to form stable complexes with various metals, spanning transition and non-transition, lanthanides, and actinides, has been pivotal in advancing coordination chemistry. Schiff base ligands are adept at stabilizing metals in different oxidation states, facilitating a variety of catalytic reactions using Schiff base metal complexes [6]. The growing interest in transition metal complexes of Schiff bases is attributed to their function within biological systems, exhibiting antimicrobial [7], antioxidant, anti-inflammatory, anticonvulsant antiproliferative properties [8], as well as their catalytic applications [9].

Morpholine derivatives have a significant impact on the treatment of various diseases. They were shown to exhibit anti-microbial, anti-HIV, antiproliferative, analgesic, local anesthetic, and anti-inflammatory properties [[10], [11], [12]]. Morpholine-derived Schiff bases exhibit complete stability in biological systems, enabling robust and prolonged applications, representing a radical redesign of DNA [13].

4,6-diacetyl resorcinol (DAR), characterized as a bifunctional carbonyl molecule, acts as a basis for the development of various multi-binding ligands [14]. In recent years, significant focus has been directed toward exploring the coordination chemistry and biological applications of 4,6-diacetyl resorcinol [15,16]. They are extensively employed in the production of organic-metal structures. Our earlier research detailed a straightforward synthesis approach for neutral bimetallic bis-Schiff bases involving zinc and aluminum alkyls, interconnected by a central resorcinol moiety. The resulting aluminum methyl species were efficient initiators for the ε-caprolactone polymerization [17].

Assays for cytotoxicity quantify a material's capacity to harm or destroy cells. These tests are crucial in research and drug development to identify harmful substances that may be used in preclinical and clinical studies for treating various pathogens in humans and animals [18,19].

Aromatase (also known as estrogen synthase) is an enzyme complex called cytochrome P450, responsible for converting C19 androgens into C18 estrogens. Studies have shown that in controlled conditions, aromatase is effective in breast tissue, and aromatase expression is most pronounced in or around breast tumor locations. This implies that the control of aromatase by internal and external factors can impact the amount of estrogen accessible for the growth of breast cancer cells [20].

Based on these observations and considering research in this area, the present study, encouraged us to investigate the Schiff base compound (HL) coordination chemistry derived from the reaction between DAR and 4-(2-aminoethyl) morpholine with d^10^ and transition metal ions such as Zn(II), Cd(II), Cu(II), and Ni(II). The crystal structure of the ligand was investigated using X-ray diffraction. The mono Schiff base ligand and synthesized mononuclear metal complexes were characterized based on elemental analyses, molar conductivity, FT-IR, ^1^H NMR, ^13^C NMR, ESR, molar conductance measurements, mass analysis, electronic spectral studies and magnetic susceptibility measurements. To explore the biological traits of these compounds, their cytotoxic effects on the MCF-7 human breast cancer cell line were evaluated via the implementation of the colorimetric MTT assay. We additionally performed docking simulations to evaluate the compounds' cytotoxicity in vitro and studied *in silico* ADME properties to assess the drug-like characteristics of the synthesized compounds.

## 2 Results and discussion

### 2.1 Chemistry and characterizations

**Scheme 1:**
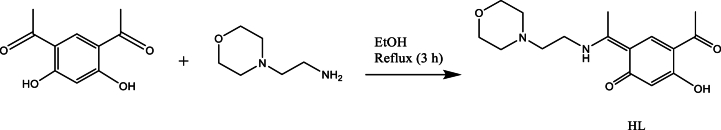
Synthesis pathway of HL.

The Schiff base, HL, (E)-4-acetyl-5-hydroxy-2-(1-((2-morpholinoethyl)iminio) ethyl)phenolate, derived from DAR and 4-(2-aminoethyl) morpholine, was prepared in a molar ratio of 1:1 according to Scheme 1. Attempts to prepare the bis-Schiff base in a ratio of 1:2 was not successful. The resultant precipitate of condensation reaction in ethanol was in good yield and the product was recrystallized through the solvent's gradual room temperature evaporation. The yellow-colored novel Schiff base ligand is stable at ambient temperature. It is soluble in both polar and non-polar solvents. Using single crystal X-ray crystallography, elemental studies, IR, ^1^H NMR, ^13^NMR, and electronic and mass spectra, the NH-form of the Schiff base was clarified.

**Scheme 2:**
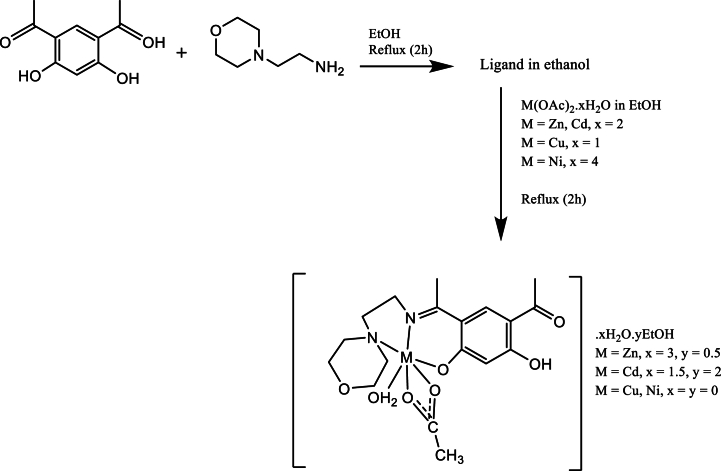
Synthesis pathway of metal complexes and their proposed structures.

Every novel complex ([M(L)(OAc)(H_2_O)].xH_2_O.yEtOH, where M = Zn, x = 3, y = 0.5; M = Cd, x = 1.5, y = 2; M = Cu or Ni, x = y = 0) were synthesized via in situ preparation of the ligand under reflux then the incorporation of the relevant salts of metal chlorides (Scheme 2). The complexes exhibited stability at ambient temperature and exhibited solubility in DMSO and DMF, but did not dissolve in methanol and ethanol. Utilizing FT-IR, elemental analysis, UV–Vis, ^1^H NMR, and mass analysis, the compounds were thoroughly characterized. The research findings indicated the octahedral structure of the Zn(II), Cd(II), Cu(II), and Ni(II) complexes. The non-electrolytic nature of these compounds was determined by measuring their molar conductance in DMSO (1 × 10^−3^ M) at 25 °C, with values ranging from 2.16 to 5.76 Ω^−1^ cm^2^ mol^−1^ [19]. Additionally, the melting point values exceeded 230 °C. The Schiff base and complexes' data (Table 1) match the ligand's structure shown in Scheme 1 and the complexes' structures outlined in Scheme 2.

**Table 1:** Analytical data and physical properties of ligand HL and its complexes.

Compounds	Empirical formula	Mol. wt. (g/mol)	Colour	m.p. (°C)	Yield (%)	Elemental found (calc.)			Ω (Ohm^−1^cm^2^mol^−1^)
						C	H	N	
HL	C_16_H_22_N_2_O_4_	306.35	Yellow	200	57.9	62.4 (62.73)	7.3 (7.24)	9.2 (9.14)	1.89
[Zn(L)(OAc)(H_2_O)]. 3H_2_O.0.5 EtOH	C_19_H_35_ZnN_2_O_10.5_	524.88	Pale gray	234	92.44	43.2 (43.48)	6.3 (6.72)	5.1 (5.34)	3.77
[Cd(L)(OAc)(H_2_O)]. 1.5H_2_O.0.2EtOH	C_22_H_41_CdN_2_O_10.5_	613.98	Pale gray	245	70.31	43.8 (43.04)	6.2 (6.73)	4.3 (4.56)	2.16
[Cu(L)(OAc)(H_2_O)]	C_18_H_28_CuN_2_O_8_	445.10	Blue	230	60.70	46.5 (46.60)	6.2 (6.08)	6.3 (6.04)	2.17
[Ni(L)(OAc)(H_2_O)]	C_18_H_26_NiN_2_O_7_	440.11	Light blue	240	53.77	48.7 (49.01)	5.8 (5.94)	6.1 (6.35)	5.76

#### 2.1.1 XRD analysis of HL

**Fig. 1:**
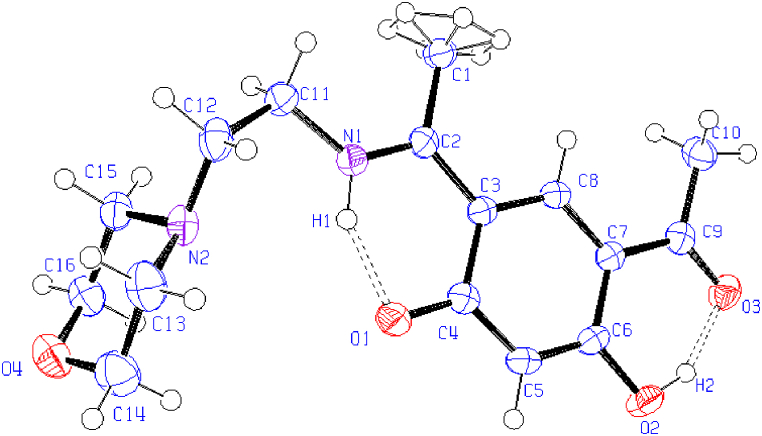
A view of (HL) displaying displacement ellipsoids with a 50 % probability. A hydrogen bond is indicated by the dashed line**.**

**Fig. 2:**
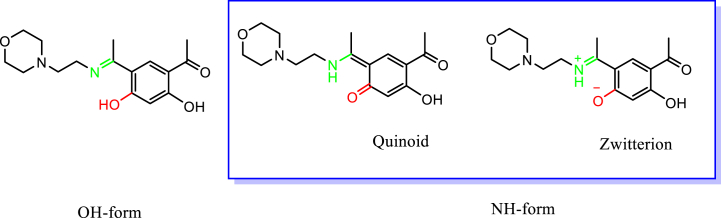
Tautomeric forms of HL.

**Fig. 3:**
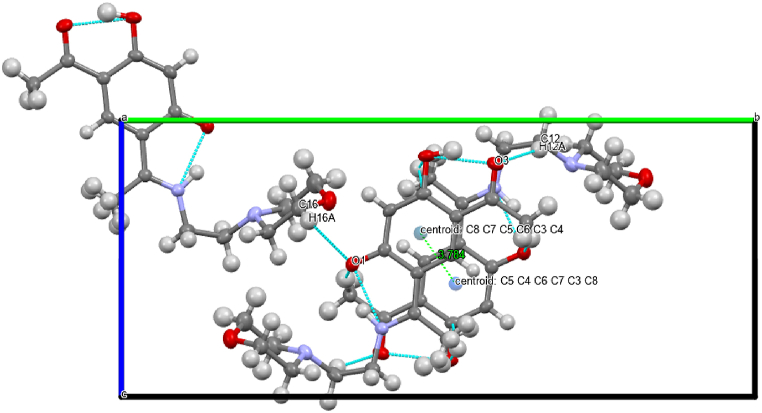
A portion of the crystal packing showing one stack of molecules along an axis showing intermolecular, intramolecular hydrogen bonding and Cg1···Cg1 distance (dashed lines).

X-ray diffraction study of HL ((*E*)-4-acetyl-5-hydroxy-2-(1-((2-morpholinoethyl) iminio)ethyl)phenolate) compound has been carried out. Fig. 1 displays the molecular structure with atom numbering, and Table S1 presents the data that were collected. Table S2 compiles a number of geometric parameters, such as bond length and bond angle. Furthermore, the geometric factors associated with intramolecular and intermolecular hydrogen bonding are shown in Table S3. The crystal structure of the compound, crystallized in a Monoclinic, space group *P*2_1_/*n*, with Z = 4 for the formula unit, ‘C_16_H_22_N_2_O_4_’. Schiff bases can be characterized by two intra-molecular hydrogen bonding types, either as N-H…O hydrogen bonds in the keto-amine form (NH form) [21] or as N…H-O hydrogen bonds in the phenol-imine form (OH form) [22,23]. The current X-ray analysis reveals that HL is a Schiff base and predominantly present in the solid state in the NH form. A perspective view of HL shows intramolecular (N1-H1···O1) hydrogen bond, involving the N-H group and the phenolic O atom, generates a six-membered ring, identified as S (6) (Fig. 1) [24]. However, uncertainties persist regarding the actual structure, whether zwitterion or quinoid, for the NH tautomer (Fig. 2). Firstly, the N1–C2 bond (1.3055 (18) Å) appears shorter than the azomethine C=N bond observed in our previously reported neutral bimetallic bis-Schiff base aluminum methyl complex [N1-C5, 1.3085 (16) Å] [17]. Secondly, the O1–C4 bond (1.2778 (17)) is considerably longer than the standard C=O bond in the acetyl group (O3-C9 = 1.2530 (17) Å) and shorter than the C–O bond in phenol (C6-O2 = 1.3559 (17) Å). These observations suggest that the C–N and C–O bonds exhibit intermediate characteristics between single- and double-type bonds. Similarly, the C2–C3 and C3–C4 bonds (1.4462 (19) and 1.4606 (19) Å displayed a character that was halfway between single and double bonds (the average of the C−C bond in ethane (1.534 Å) and ethene (1.337 Å)). The aforementioned observations indicate that compound HL would have characteristics halfway between zwitterion and quinoid, such as a delocalized ionic property analogous to a non-classical ion [25]. The crystal structure is stabilized by intermolecular, intramolecular hydrogen bonding and a weak stacking π-π interaction [Cg1···Cg1 = 3.784 Å]; where Cg1 is the centroid of the C8-C7-C5-C6-C3-C4 ring of the resorcinol ring system as shown in Fig. 3.

#### 2.1.2 NMR spectral studies (^1^H and ^13^C NMR spectra)

The signals in the ligand's spectrum of ^1^H NMR in DMSO-d_6_ are described in Table S4 and shown in Fig. S1. A fairly broad signal at *δ* 12.64 ppm was observed for the phenolic OH group. The resorcinol ring protons are displayed at *δ* 8.19 and *δ* 5.84 ppm. Ethylenic moiety protons (-CH_2_-) are seen at *δ* 3.76 and *δ* 2.63 ppm (Triplet). The peak at *δ* 3.59 ppm is attributed to -O-CH_2_ in the morpholine moiety. The peak at *δ* 2.57 ppm, which is a triplet, has been allocated to (-N-CH_2_) in the morpholine ring. The broad -NH proton resonated at *δ* 2.46. The six protons of the two methyl groups resonated at *δ* 2.10, two separate peaks were expected, however, the two peaks were superimposed on each other. The ligand's^13^C NMR spectrum was taken in DMSO-d_6_, and the spectral information validates the findings from the ^1^H NMR spectrum. In the ^13^C NMR spectrum, the quantity of signals is equivalent to the number of magnetically fourteen different carbon atoms in HL (Fig. S2). The acetyl carbon atom is identified at *δ* 207.27 ppm, and the carbon-oxygen peak (intermediate between C-O and C=O) appeared at *δ* 202.39, while the signal at *δ* 179.32 can be assigned to phenolic C-OH. The peak at 175.44 is due to carbon-nitrogen signal (intermediate between C-N and C=N). Peaks in the region *δ* 166.35–105.80 ppm are due to aromatic carbons. Methyl carbons are seen at 26.50 and 15.30 ppm and ethylinc and morpholine ring carbons were observed in the region 66.87–31.16 ppm. Therefore, the formation of the ligand 10.13039/100023110HL as suggested and supported by single crystal x-ray diffraction is confirmed by the ^1^H and ^13^C 10.13039/501100004182NMR spectra.

The ^1^H NMR spectrum of the Zn(L)(OAc)(H_2_O)].H_2_O.1.5EtOH and [Cd(L)(OAc)(H_2_O)]0.1.5H_2_O.2EtOH complexes in DMSO-d_6_ displays signals that are comparable to those seen with HL (Figs. S3 and S4 and Table S4) The absence of a signal due to the NH proton confirm the engagement of the azomethine nitrogen atom in bonding to the metal ion occurs through deprotonation. The signals at *δ* 1.21 and *δ* 1.22 for the Zn(II) and Cd(II) respectively are consistent with the binding of the acetate ligand to the metal center in solution [26,27]. For the Cd(II) complex, the existence of dual peaks for each signal suggests a potential equilibrium among various conformations in the solution, leading to the non-equivalence of protons in the two halves of the molecules [22].

#### 2.1.3 Infrared spectra and coordination mode

The complexes' spectra and the free ligand's FTIR spectrum were compared (Figs. S5–S9). Table 2 lists the most important infrared bands and their corresponding assignments. The spectrum of Schiff base shows a wide band at 3418 cm^−1^ that indicates the presence of a strongly hydrogen-bonded OH group of resorcinol (O−H∙∙∙O) and the internal hydrogen bonding vibration (O^−^ … H–N^+^) [25]. Broad bands in the 3435–3441 cm^−1^ range were observed for all metal complexes, suggesting the existence of coordinated molecules of water [28]. The absorption band appearing in the ligand spectrum at 1638 cm^−1^ is attributed to v (C=NH^+^) stretching vibration. In the complexes, the azomethine (C=N) frequency shows a downfield shift suggesting coordination through the azomethine nitrogen atom. The absorption bands within the range of 1630–1645 cm^−1^ may be ascribed to the acetyl C=O group stretching vibrations. Aromatic rings stretching vibration of v (C=C) appears in the vicinity1409-1537 cm^−1^. The ligand's vC-O (phenolic) stretching frequency is observed at 1280 cm^−1^ and shifts to a lower or higher frequency band in the complexes within the 1339–1382 cm^−1^ range, indicating phenolic oxygen bonding. Novel bands at 420-466 cm^−1^ and 579-674 cm^−1^, representing the v (M−O) and v (M−N) stretching, respectively, provide additional evidence for the M(II) atoms bonding to the ligand through the nitrogen and oxygen atoms [29]. Consequently, -C=N and -CO are shown to be the coordination sites of the metal ion in the IR spectrum data.

**Table 2:** Characteristics of the Schiff base's and all metal complexes' infrared bands (cm^−1^).

Compound	*v(*OH)/*v*H_2_O)	v (C=O)	v (C=N)	*v(*C=C)	v (C-O)	v (M − O)	_v_ (M − N)	*v* (COO^−^) (bidentate OAc−)
HL	3418	1638	1617	1537	1380	–	–	–
[Zn(L)(OAc)(H_2_O)].3H_2_O.0.5 EtOH	3440	1634	1598	1432	1339	672	563	1316 *v*_as_ (COO^−^), 1252 *v*s (COO^−^)
[Cd(L)(OAc)(H_2_O)]0.1.5H_2_O.2 EtOH	3441	1645	1591	1490	1371	579	457	1339 *v*_as_ (COO^−^), 1258 *v*s (COO^−^)
[Cu(L)(OAc)(H_2_O)]	3435	1614	1568	1409	1385	662	420	1331 *v*_as_ (COO^−^), 1270 *v*s (COO^−^)
[Ni_2_(L)(OAc)(H_2_O)]	3436	1630	1575	1425	1382	674	466	1319 *v*_as_ (COO^−^), 1252 *v*s (COO^−^)

The chelating bidentate CH_3_COO^−^ group in the complexes was verified by the emergence of additional bands in the 1316–1339 and 1252-1270 cm^−1^ ranges. These two bands result from, respectively, v_s_ (COO^−^) and v_as_ (COO^−^). The two bands' separation, Δv = (*v*_*as*_*-v*_*s*_) = 61-81 cm^−1^, is similar to the values reported for the acetate group's bidentate nature [14].

#### 2.1.4 Solvent effect on the electronic absorption of HL, magnetic moments and electronic spectral data of complexes

The UV–vis spectra depicting the maximum wavelengths of electronic absorption for the HL ligand in solvents of varying polarities are illustrated in Fig. S10. In this depiction, HL exhibits three absorption bands between the following wavelength ranges 230–300 nm, 295–350 nm, and 375–550 nm. Notably, the first and second bands appear intense, while the third band is both weaker and broader. The broad character of the third band suggests charge transfer within the molecule. The first electronic transition band is attributed to the delocalization of electrons in the resorcinol ring, representing the π-π* electronic transition. The second absorption band, sensitive to the solvent, results from electronic transitions in the C=NH group, involving conjugation between the resorcinol ring and the C=NH group. The third absorption, weak and broad, is identified as an n-π* transition, indicating delocalization with a lone-pair electron. The electronic absorption bands are susceptible to changes in solvent polarity, although these changes do not occur consistently and instead tend to cause a bathochromic shift [10].

**Fig. 4:**
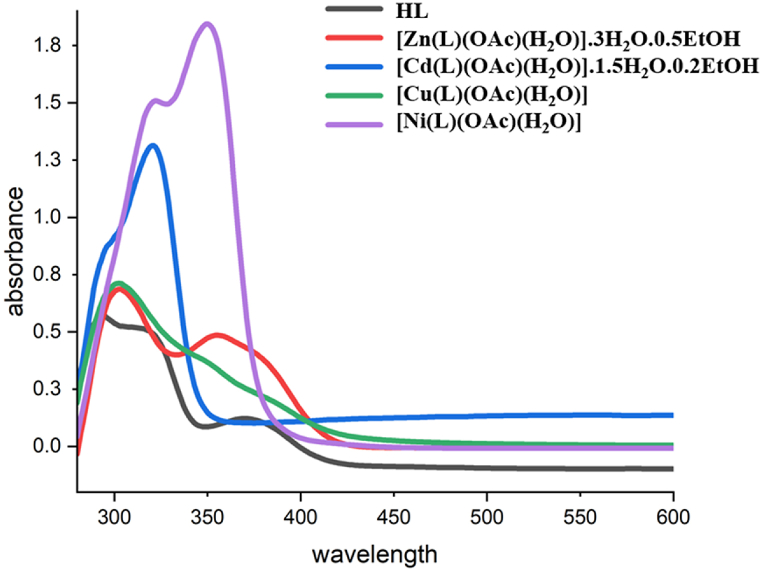
The HL and its metal complexes' UV–vis spectrum in DMSO at 0.0001 M.

Magnetic moments and electronic absorption spectra of the metal complexes provided information on their geometry, as presented in Table S5 (Fig. 4) utilizing DMSO as a solvent. In the spectra of all metal complexes, the bands of absorption associated with π-π* and n–π* transitions, originally observed in the ligand's spectrum, have experienced shifts owing to the ligand's coordination with metal ions. The absence of d-d bands in the Zn(II) and Cd(II) complexes aligns with expectations for a d^10^ system, and their diamagnetic nature. The octahedral geometry of these complexes is determined by conductance, analytical and spectral data. The Cu(II) complex's electronic spectrum exhibits a wide band, roughly centered at 700 nm, revealing the ^2^E_g_→^2^T_2g_ transition, which is an indication of an octahedral geometry centered on the Cu(II) ion. The 1.77 B M. magnetic moment value further supports the electronic findings and is compatible with a single unpaired electron in the 3d shell of the 3 d^9^ configuration [30,31].

The (UV–Vis) spectrum of Ni(II) complex displays two weak broad peaks in the visible region at 625 nm and 770 nm were attributed to (^3^A_2g_(F) _→_^3^T_1g_(P)) and (^3^A_2g_(F) _→_^3^T1g(F))) d–d transitions, the observed spectral features confirm the octahedral structure surrounding the nickel (II) ion. The magnetic moment value of 3.12 BM is compatible with a high-spin octahedral geometry [31].

#### 2.1.5 Mass spectra

To compare the stoichiometric composition of HL and all metal complexes, the ESI mass spectra were utilized and are listed in Table S6 (Figs. S11–S15). A main molecular ion peak was visible in the ligand (HL) spectrum at *m*/*z* 307.17 which corresponds to the protonated species [HL + H]^+^ (C_16_H_23_N_2_O_4_) peak as the determined *m*/*z* was 306.35. In the Zn(II) complex mass spectrum, a peak appears at *m*/*z* = 525.073, representing the [M]^+^ peak, while at *m*/*z* = 470.022 the peak matches [M-3H_2_O]^+^. The mass analysis, combined with elemental analysis, substantiates the complex's structure and validates that [Zn(L)(OAc)(H_2_O)].3H_2_O.0.5EtOH is the formulae of metal chelates. Within the Cd(II) complex mass spectrum, a peak is noted at *m*/*z* = 613.329, indicative of the [M]^+^ ion. Another peak at 513.228 corresponds to [M-2EtOH - 0.5H_2_O]^+^, and the prominent peak at *m*/*z* = 307.19 represents [HL + H]^+^. Mass analysis, coupled with elemental analysis, substantiates the complex's structure and affirms the stoichiometry of metal chelates as [Cd(L)(OAc)(H_2_O)]0.1.5H_2_O.0.2EtOH. A peak at *m*/*z* = 441.14 in the Cu(II) complex mass spectrum indicates [M+4H]^+^. The main peak [M − (OAc) - (H_2_O)]^+^ corresponds to *m*/*z* = 368.08. The complex's structure is supported by mass analysis combined with elemental analysis, which also validates the stoichiometry of metal chelates as Cu(L)(OAc)(H_2_O). The mass spectrum of the Ni(II) complex shows a peak at *m*/*z* = 436.142, which suggests [M + 4H]^+^ peaks. The peak associated with [M − (OAc) - (H_2_O)]^+^ is located at *m*/*z* = 363.089. The peak at *m*/*z* = 307.169 corresponds to [HL + H]^+^. The complex's structure is supported by mass analysis combined with elemental analysis, which also validates that the stoichiometry of metal chelates' as Ni(L)(OAc)(H_2_O).

#### 2.1.6 ESR spectrum of Cu(II) complex

The Cu(II) complex's solid state ESR spectrum was examined at 300 K to provide further insight into the arrangement of elements surrounding the Cu metal ion. Diphenylpicrylhydrazyl (DPPH) was employed as a g-marker to determine the g-values. There is just one strong absorption band visible in the spectrum at a high field at 2.185 (Fig. S16), which, because of the molecules' tumbling motion, is isotropic, or due to similar coordination bond distances for all six Cu–L of the octahedron [32,33]. This arises from improper coordination through the non-magnetic (dz^2^) Cu orbital, resulting in a negligible or absent exchange interaction. The ESR-calculated magnetic susceptibility of the Cu(II) complex is 1.89 Bohr magnetons (BM), suggesting one unpaired electron is present, which verifies the complex's mononuclear character. The ESR spectrum's lack of a half-field signal at 1600 G lends more credence to this, attributed to ms = ±2 transitions, eliminating the possibility of any Cu–Cu interaction [34].

### 2.2 Cytotoxic activity

**Fig. 5:**
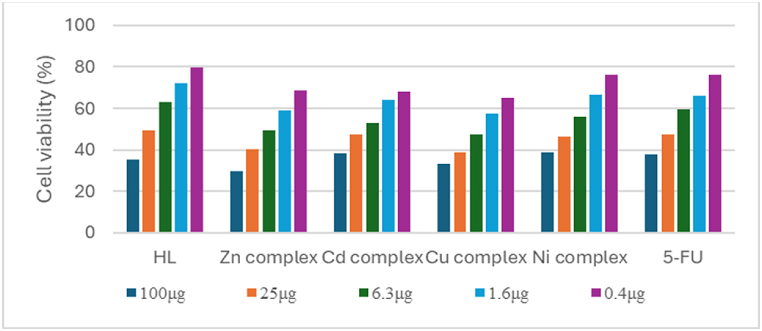
The percentage of cell viability after treatment of MCF-7 cells with different weights of Samples.

The in vitro cytotoxic properties of the newly produced HL and the metal complexes were assessed utilizing the human breast cancer cell line MCF-7 and the MTT colorimetric technique (Fig. 5; Table 3). Among the findings are IC_50_ readings, representing the concentration of a compound (in μM) necessary to cause 50 % cell death in comparison to the control culture. 5-FU served as a reference. Notably, the Schiff base HL demonstrated sensitivity in MCF-7 cells, having an IC_50_ value of 69.01 ± 1.21 μM, higher than all metal complexes but lower than 5-FU. Overall, all the complexes exhibited superior effectiveness against the human cancerous breast cell line in comparison to the Schiff base ligand and 5-FU, as evidenced by the complexes' lower IC_50_ values. It was hypothesized that the tumor-inhibiting capability of these compounds was attributed to their chelating properties [7]. The most active of the tested compounds was the Cu(II) complex against MCF-7 cells with IC_50_ values of 10.51 ± 0.27 μM, the zinc(II) complex came next and then the cadmium complex. Meanwhile, the Ni(II) complex exhibited the lowest toxicity with an IC_50_ value of 38.37 ± 0.97 μM.

**Table 3:** In vitro antiproliferative activities of HL and the metal complexes against MCF-7 human tumor cell line.

Compound	Cytotoxicity IC_50_^a^ (μM)	SD ±
HL	69.01	1.21
[Zn(L)(OAc)(H_2_O)].3H_2_O.0.5 EtOH	10.77	0.33
[Cd(L)(OAc)(H_2_O)]0.1.5H_2_O.0.2EtOH	20.75	0.73
[Cu(L)(OAc)(H_2_O)]	10.51	0.27
[Ni(L)(OAc)(H_2_O)]	38.37	0.97
5-FU^b^	138.7	1.04

a The concentration that causes a 50 % cell proliferation inhibition.

b Used as a positive control.

### 2.3 Molecular docking study

**Fig. 6:**
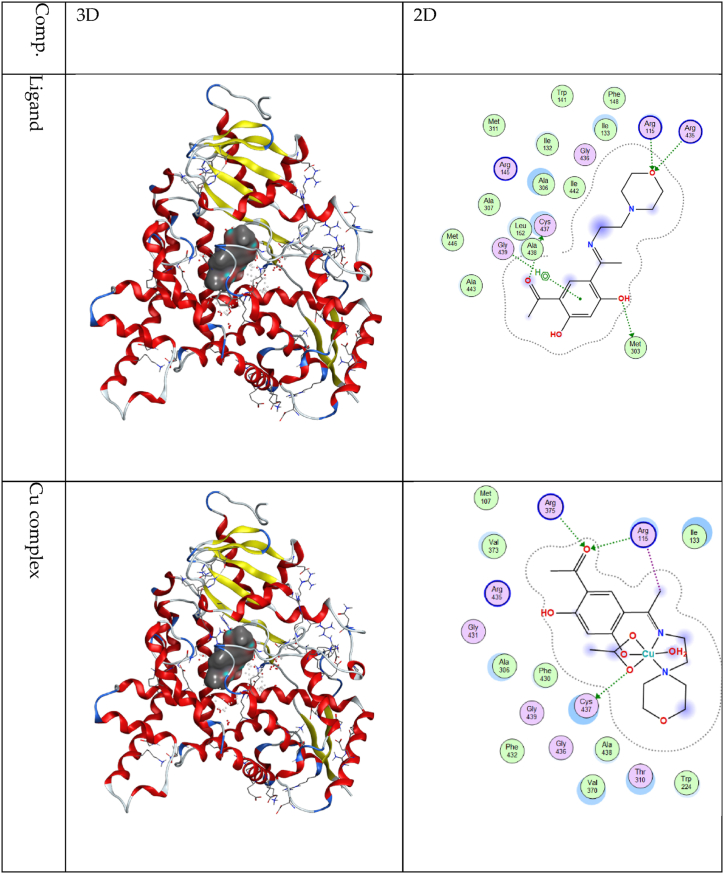
2D, and 3D Docking interaction of the ligand and Cu complex and 3eqm as breast cancer protein.

The synthesized ligand and its metal complexes underwent screening against the "3eqm" protein associated with breast cancer (Tables S7 and S8 and Fig. S16). This protein functions as the enzyme known as aromatase cytochrome P450, which is in charge of the production of estrogens [[35], [36], [37]]. Scores for docking the ligand and every complex with the "3eqm" protein were found to align with the experimental data presented in Table S7. Copper was the complex that showed the highest docking score with the selected protein in comparison with the ligand and other complexes (Fig. 6). The docking scores were as follows: −8.13 kcal/mol due to a developed hydrogen bond between one atom of oxygen from the acetate group and one atom of sulfur of CYS 437, -3.02 kcal/mol due to the ligand's carbonyl group oxygen atom and the neighboring nitrogen atom of NH1 ARG 115, -2.30 kcal/mol due to a hydrogen bond between the same oxygen atom of the ligand's carbonyl group and the nitrogen atom of NH2 ARG 115, -2.00 kcal/mol because the nitrogen atom of NH2 ARG 115 and the same oxygen atom of the ligand's carbonyl group have formed a hydrogen bond, and −3.00 kcal/mol due to an ionic interaction between the methyl group carbon atom of the ligand and the nitrogen atom of NH2 ARG 115.

### 2.4 In silico ADMET studies

**Fig. 7:**
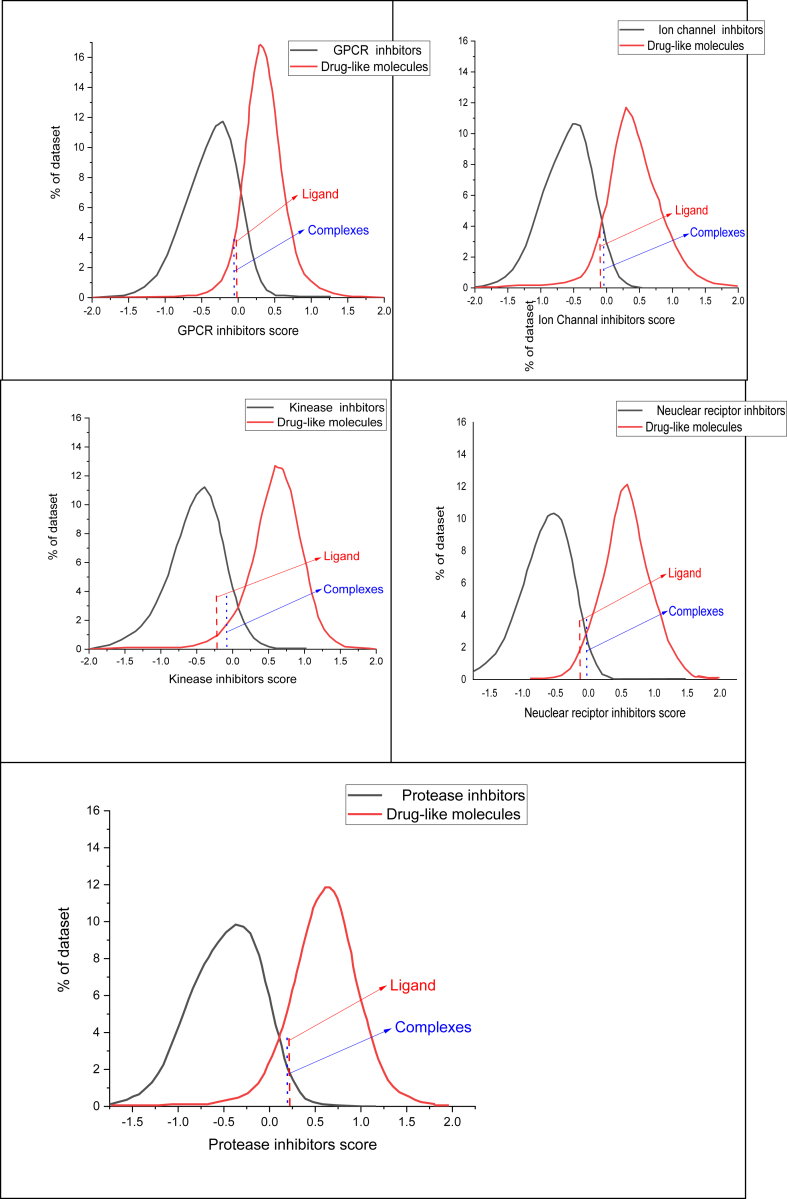
Graphs depicting the ADME profiles and drug-likeness scores of the ligand and its metal complexes.

Our investigation verifies that both the synthesized Ligand and its complexes adhere to Lipinski's criteria, as detailed in Table 4, Table 5. Their determined bioactivity scores fall between −0.46 and 0.13, and their molecular weights are under 500, suggesting a high similarity to known drugs. Furthermore, the ligands and metal complexes display logP values of 1.83, −5.98, −6.03, −5.93, and −6.03, showing superior permeability and absorption qualities, which are crucial in pharmaceutical chemistry. The favorable drug-like characteristics of these compounds are further supported by the drug-likeness model score, which is shown in Fig. 7.

**Table 4:** Lipinski properties of the compounds.

	Ligand	Ni Complex	Cu Complex	Zn Complex	Cd Complex
miLogP	1.83	−5.98	−6.03	−5.93	−6.03
TPSA	82.36	100.62	100.62	100.62	100.62
Natoms	22	28	28	28	28
MW	306.36	442.11	446.97	448.81	495.83
nON	6	9	9	9	9
nOHNH	2	3	3	3	3
nviolations	0	0	0	0	0
nrotb	5	1	1	1	1
volume	287.18	368.09	368.09	368.09	368.09

**Table 5:** ADME properties of the compounds.

	Ligand	Ni complex	Cu complex	Zn complex	Cd complex
***GPCR ligand***	*−0.17*	*0.04*	*0.04*	*0.04*	*0.04*
***Ion channel modulator***	*−0.25*	*−0.10*	*−0.10*	*−0.10*	*−0.10*
***Kinase inhibitor***	*−0.46*	*−0.19*	*−0.19*	*−0.19*	*−0.19*
***Nuclear receptor ligand***	*−0.16*	*−0.12*	*−0.12*	*−0.12*	*−0.12*
***Protease inhibitor***	*−0.33*	*−0.07*	*−0.07*	*−0.07*	*−0.07*
***Enzyme inhibitor***	*−0.09*	*0.13*	*0.13*	*0.13*	*0.13*

## 3 Experimental section

### 3.1 Materials and instrumentation

We utilized solvents and reagents that were bought commercially without additional purification. 4,6- diacetylresorcinol and 4-(2-aminoethyl) morpholine were purchased from Sigma-Aldrich (Burlington, Massachusetts, USA). Zinc acetate dihydrate (Zn(OAc)_2_.2H_2_O), Cadmium acetate dihydrate (Cd(OAc)_2_.2H_2_O), Copper acetate monohydrate (Cu(OAc)_2_.H_2_O), Nickel acetate tetrahydrate (Ni(OAc)_2_.4H_2_O) were procured from BDH chemicals (London, UK). Thermo Flash 2000 CHN Elemental analyzer was employed for elemental analyses. A PerkinElmer FT-IR Spectrometer was used to record the infrared spectra of the Schiff base ligand, and its metal complexes in KBr pellets. The spectra covered the 4000–400 cm^−1^ range. A Brucker Ascend Spectrophotometer operating at 850 MHz was used to obtain the ^1^H and ^13^C NMR spectra. Employing a MultiSpec-1501 UV–VIS spectrophotometer, electronic spectra for the ligand and its complexes in the 200–800 nm range were obtained in DMSO. These spectra were obtained using a 1.0 cm quartz cell at ambient temperature. The continuous wave Bruker EMX PLUS spectrometer (Bruker BioSpin, Rheinstetten, Germany) was used for paramagnetic resonance (EPR) analysis in the solid state. The Sherwood Scientific Magnetic susceptibility balance was used to measure magnetic susceptibility at room temperature. An OHAUS-STARER 3100C conductance detector was used to measure molar conductance at room temperature using a 0.001 M complex solution in DMSO.

### 3.2 Crystal structure determination of HL

The crystallographic information from single-crystal X-ray diffraction was collected at a temperature of 150 K, employing a CCD diffractometer, Bruker APEX-II. A radiation source of MoKα monochromated with graphite with a wavelength (λ) of 0.71073 A was employed for the analysis, along with the w-scan method. Initially, the structures were dealt with SHELXT through direct approaches, and then, further refinement was done using full matrix least squares method within the OLEX2 program suite, where non-hydrogen atoms were treated with anisotropic displacement parameters. From another Fourier map, the hydrogen atoms were identified and processed isotropically. Bruker APEX2 was used for data collection, Bruker SAINTl for cell refinement, and Bruker SAINT for data reduction. The crystallographic tool PLATON facilitated the structure analysis, while structural analysis and the results were presented using MERCURY and ORTEP-3 programs.

### 3.3 Preparation of the ligand (E)-4-acetyl-5-hydroxy-2-(1-((2-morpholinoethyl)iminio)ethyl)phenolate (HL)

Upon the full dissolution of the 4,6-diacetylresorcinol (0.097 g, 0.5 mmol) in ethanol (10 mL), an equimolar quantity of 4-(2-aminoethyl) morpholine (0.065 g, 0.5 mmol) in ethanol was added (10 mL). As a catalyst, the reaction mixture acquired three drops of acetic acid anhydride added to it. After that, the mixture was allowed to reflux for 3 h before being permitted to come to ambient temperature. The Schiff base, called HL, was then obtained by gathering the precipitate that resulted and washing it with ethanol. The dissolved compound in ethanol was slowly evaporated resulting in crystals of HL. Color: yellow; yield: 57.90 % (0.09g); m.p.: 200 °C. Anal. calc., (C_16_H_22_N_2_O_2_) C, 62.7; H, 7.2; N, 9.1 found, C, 62.6; H, 7.3; N, 9.2 ^1^H NMR (850 MHz, DMSO, ppm) *δ* 12.6 (v.b. s, 1H, -OH), 8.19 (s, 1H, H_r-ring_), 5.84 (s, 1H, H_r-ring_), 3.76 (t, 2H, *J* = 6.63 Hz, (-N-CH_2_) in ethylenic moiety), 3.60 (t, 4H, *J* = 4.08 Hz, -O-CH_2_), 2.63 (t, 2H, *J* = 6.63 Hz, (-CH_2_-N) in ethylenic moiety), 2.56 (t, 4H, *J* = 3.50 Hz, (-N-CH_2_) in morpholine ring), 2.46 (bs, 1H, CH_3_C-NH), 2.09 (s, 6H, -CH_3_). ^13^C NMR (850 MHz, DMSO) *δ* 207.27 (C=O acetyl), 202.39 (C-O (or C=O)), 179.32 (C-OH), 175.44 (C-N (or C=N), 166.35 (C-Ar), 138.51 (C-Ar), 110.57 (C-Ar), 105.80 (C-Ar), (*δ* 66.87, 56.89, 53.67, 31.16, 26.50) C-ethylenic and morpholine, 26.50 (CH_3_- Methyl), 15.30 (CH_3_-Methyl).

### 3.4 Preparation of metal complexes

The following is how the metal complexes were prepared: 4,6-diacetylresorcinol (0.097 g, 0.5 mmol) was dissolved in 10 mL of ethanol and added to an ethanolic solution of 4-(2-aminoethyl) morpholine (0.065 g, 0.5 mmol) in ethanol (10 mL). The mixture was refluxed for 2 h, after which an ethanolic solution of (Zn(OAc)_2_.2H_2_O), (Cd(OAc)_2_.2H_2_O), (Cu(OAc)_2_.H_2_O) or (Ni(OAc)_2_.4H_2_O) (0.5 mmol, 10 mL) was added. The obtained solutions underwent magnetic stirring and refluxing for 2 h. After cooling, filtration was used to collect the precipitates that resulted and subjected to multiple washes with hot ethanol, then let to dry. In the presence of moisture and air, all of the complexes showed stability. Table 1 provides comprehensive details about the physical and analytical characteristics of the metal complexes and the ligand (HL).

• For [Zn(L)(OAc)(H_2_O)].3H_2_O.0.5EtOH, a solid with a light gray shade was produced.
• ; m.p.: 234 °C; yield (92.44 %, 0.12 g). ^1^H NMR (850 MHz, DMSO, ppm).
• For [Cd(L)(OAc)(H_2_O)]0.1.5H_2_O.0.2EtOH, a solid with a light gray shade was produced; m.p.: >245 °C; yield (70.31 %, 0.22 g). ^1^H NMR (850 MHz, DMSO, ppm).
• For [Cu(L)(OAc)(H_2_O)], a blue solid was obtained; m.p.: >230 °C; yield (60.70 %, 0.14 g).
• For [Ni(L)(OAc)(H_2_O)], a solid with a light blue color was produced; m.p.: >240 °C; yield (53.77 %, 0.12 g).

### 3.5 Sample preparation and analysis for mass analysis

Dimethylformamide was used to dissolve the samples. Following the dissolution of the material, a 10-μL portion was diluted 1000 times in acetonitrile. There was no sign of turbidity or precipitation. Each sample was introduced to the liquid chromatography (LC) flow in a volume of 5 mL, which was made up of a mobile phase containing 100 % acetonitrile, as part of the flow injection method used for the analysis. After that, the samples were put straight into the electrospray ionization source for mass spectrometry to generate ionization. A quadrupole time-of-flight mass spectrometer equipped with an electrospray ionization (ESI) source was used for the studies. External calibration was carried out for the MS data using sodium formate cluster calibration data.

### 3.6 Viability of cell test

The MTT assay was used to evaluate the viability of the cells using mononuclear cells. This procedure is dependent on the conversion of soluble yellow tetrazolium salt into insoluble purple crystals of formazan via metabolically active cells. A mitochondrial succinate dehydrogenase enzyme, found within the mitochondria of living cells, transforms the tetrazolium salt that has been absorbed into purple formazan crystals. The resultant alteration in color is quantified utilizing an ROBONIK P2000 Elisa Reader at 570 nm. In the test, a single MCF-7 human breast cancer cell line is cultured in 96-well plates at a density of 1 × 10^5^ cells per well. Following a 24-h period, the cells went through two washes using 100 μL of serum-free media and underwent an hour-long starvation at 37 °C. After that, the cells were subjected to 48 h at different doses of the test chemical (0.4–100 μg/mL). Once the medium is removed, a serum-free medium containing 0.5 mg/mL of MTT is added. The mixture is then incubated for 4 h at 37 °C in a CO_2_ incubator. The MTT-containing medium is then removed, the cells are rinsed with PBS (200 μL), and the crystals are dissolved by adding 100 μL of DMSO. This solution is meticulously mixed through pipetting. The spectrophotometric measurement of the purple-blue formazan dye's absorbance is carried out using a microplate reader at 570 nm [38,39].

### 3.7 Molecula docking method

The molecular interaction between the synthesized ligand and its metal complexes with aromatase cytochrome P450 of the breast cancer receptor protein (PDB code = 3eqm) [36,40]was investigated through a molecular docking study. All docking analyses were conducted using the MOE 2019.102 platform. The 3D structures of the proteins were obtained from the Protein Data Bank "https://www.rcsb.org/" as PDB files after removing solvent molecules and correcting structures and charges as previously described [41]. Active sites designated as dummy atoms, representing the location of the active drug or co-crystalline ligand. Docking results generated using Triangle Matcher with rigid proteins, and the docking scores calculated using the London dG method for 30 poses, with the best five poses selected. The computer specifications for this study included Windows 10 and an Intel(R) Core(TM) i7-8550U CPU @ 1.80 GHz 1.99 GHz.

### 3.8 In silico ADME

The compounds underwent analysis using the Molinspiration program in SMILES format to assess their drug-likeness based on Lipinski's rule of five [42]. This rule evaluates characteristics related to ADME for determining drug-like properties. It indicates that compounds with a logP value below 5, a molecular mass under 500, and fewer than 5 H-bond acceptors and donors are likely to exhibit bioactivity [43,44].

## 4 Conclusions

In this investigation, we prepared a new NH-form of resorcinol-based Schiff Base and performed a comprehensive examination of its crystallographic data. Furthermore, we prepared and examined complexes of this Schiff base involving Zn(II), Cd(II), Cu(II), and Ni(II) employing a variety of methods, including mass spectrometry, elemental analysis, infrared spectroscopy, and magnetic measurements. The findings unequivocally reveal the bidentate nature of the mono-Schiff base ligand. The metal-to-ligand stoichiometry for all these complexes is consistently 1:1. Electronic and ESR spectral investigations suggest an octahedral geometry for each complex. Upon assessing the cytotoxicity of these metal complexes on MCF-7 cancer cells, the [Cu(L)(OAc)(H_2_O)] and [Zn(L)(OAc)(H_2_O)].3H_2_O.0.5 EtOH complexes have a significantly increased cytotoxic effect, with IC_50_ values of 10.51 ± 0.27 and 10.77 ± 0.33 μM respectively. The IC_50_ value of the Cu(II) and Zn(II) complexes are significantly lower than that of 5-FU, the conventional cancer drug (IC_50_ = 138.7 ± 1.04 μM)). The Molecular Docking explains the highest activity of the Copper complex according to its highest docking score and its hydrogen bonding interaction with the breast cancer receptor. The drug likeness model score by ADME, reinforces the favorable drug-like attributes of these compounds.

## Data availability statement

Data will be available upon reasonable request from the corresponding author. Crystallographic data associated with this study has been deposited at the Chemical Crystallographic Data Centre under the accession number CCDC 2369087.

## Funding

This research received no external funding.

## Declaration of competing interest

The authors declare that they have no known competing financial interests or personal relationships that could have appeared to influence the work reported in this article.
