# Supplementary material for: Exploring novel NH-form resorcinol-based schiff base and its metal complexes: Synthesis, characterization, cytotoxic activity, molecular docking and ADME studies
Source: Heliyon. 2024 Sep 7;10(18):e37385. doi: 10.1016/j.heliyon.2024.e37385 (PMC11416489; doi:10.1016/j.heliyon.2024.e37385)
Supplement: Multimedia component 1 [file mmc1.docx]

**Supplementary Materials**

**Exploring Novel NH-Form Resorcinol-Based Schiff Base and its Metal Complexes: Synthesis, Characterization, Cytotoxic Activity, and Molecular Docking Study"**

**Elham Aazam^1^ *, Maryam Majrashi^1^ and Mostafa A. Hussien^1,2^**

^1^Department of Chemistry, Faculty of Science, King Abdulaziz University, Jeddah P.O. Box 23622, Saudi Arabia

^2^Department of Chemistry, Faculty of Science, Port Said University, Port Said, 42521, Egypt

***** Correspondence: eazam@kau.edu.sa


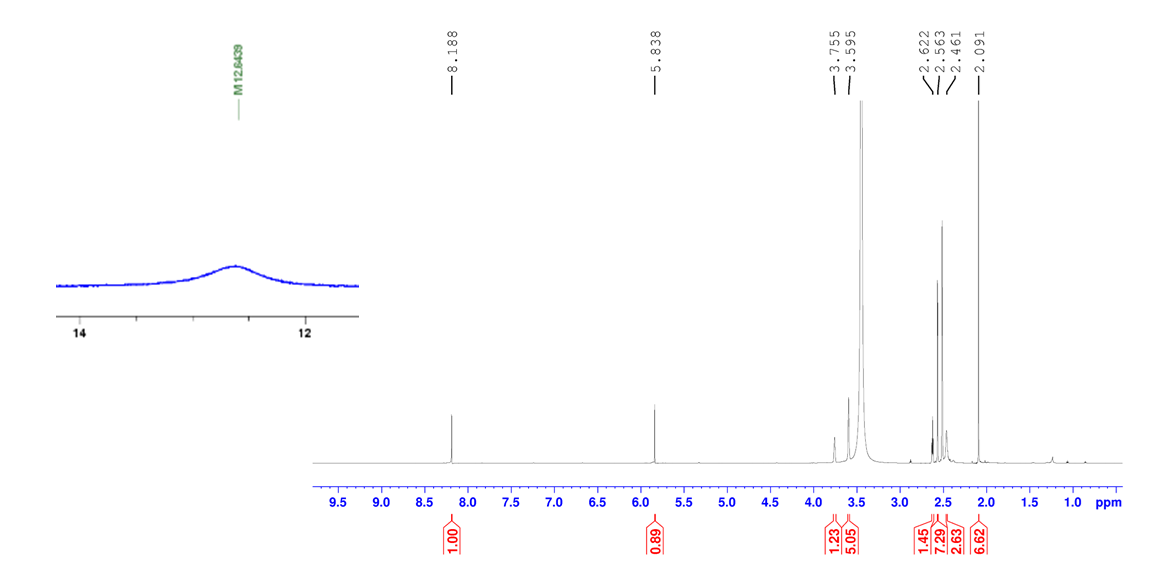


**Figure S1.** ^1^H NMR spectrum of HL.


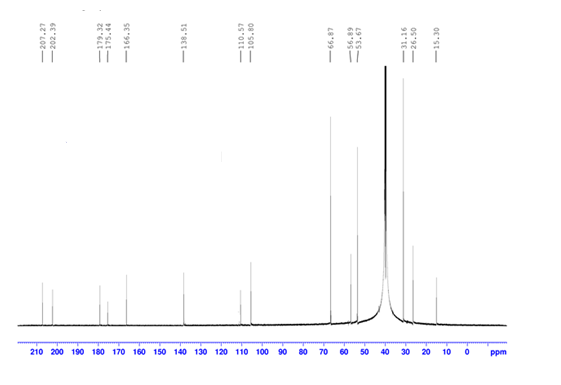


**Figure S2.** ^13^C NMR Spectrum of the ligand HL.

**
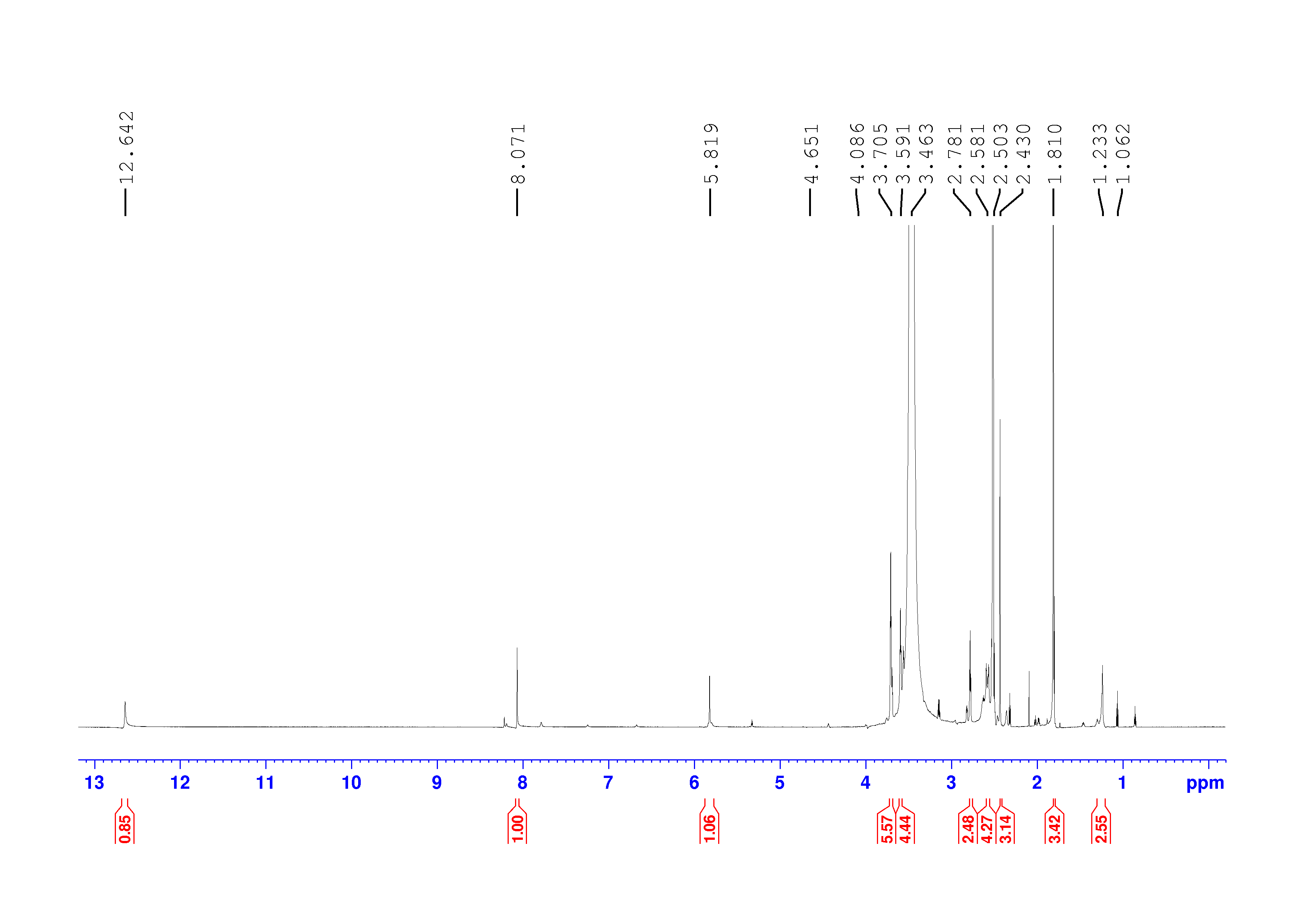
**

**Figure S3** ^1^H NMR Spectrum of [Zn(L)(OAc)(H_2_O)].3H_2_O.0.5EtOH

**
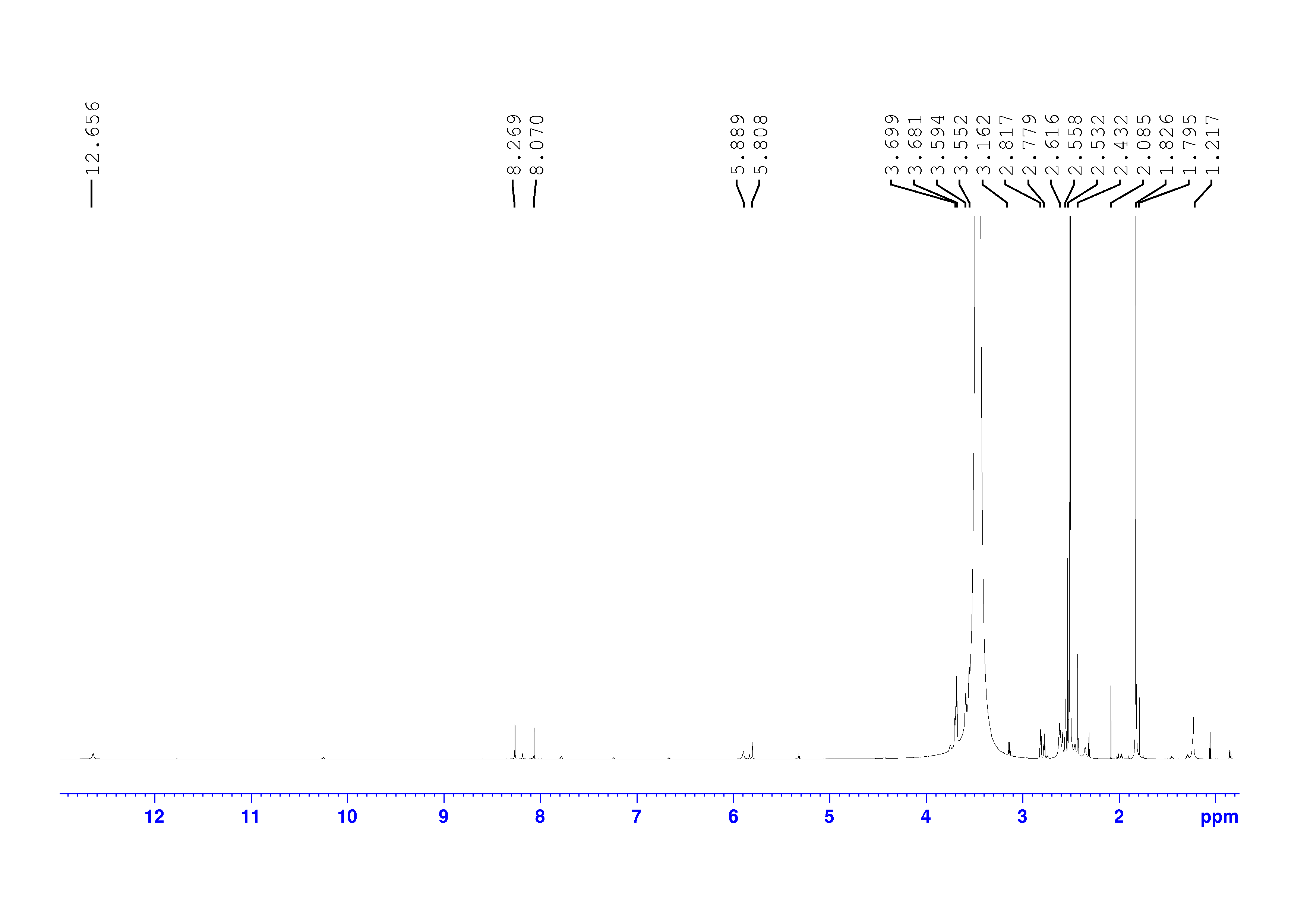
**

**Figure S4** ^1^H NMR Spectrum of [Cd(L)(OAc)(H_2_O)].1.5H_2_O.2EtOH


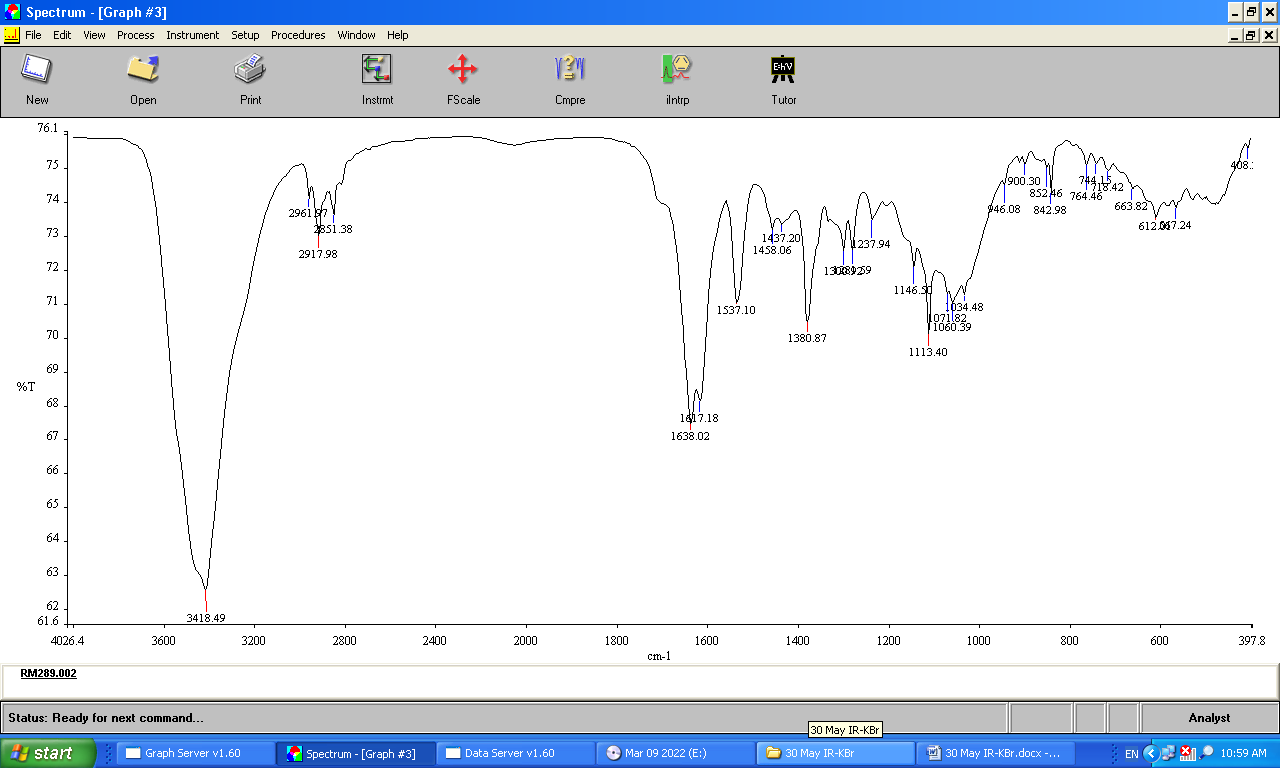


**Figure S5** IR spectrum of HL


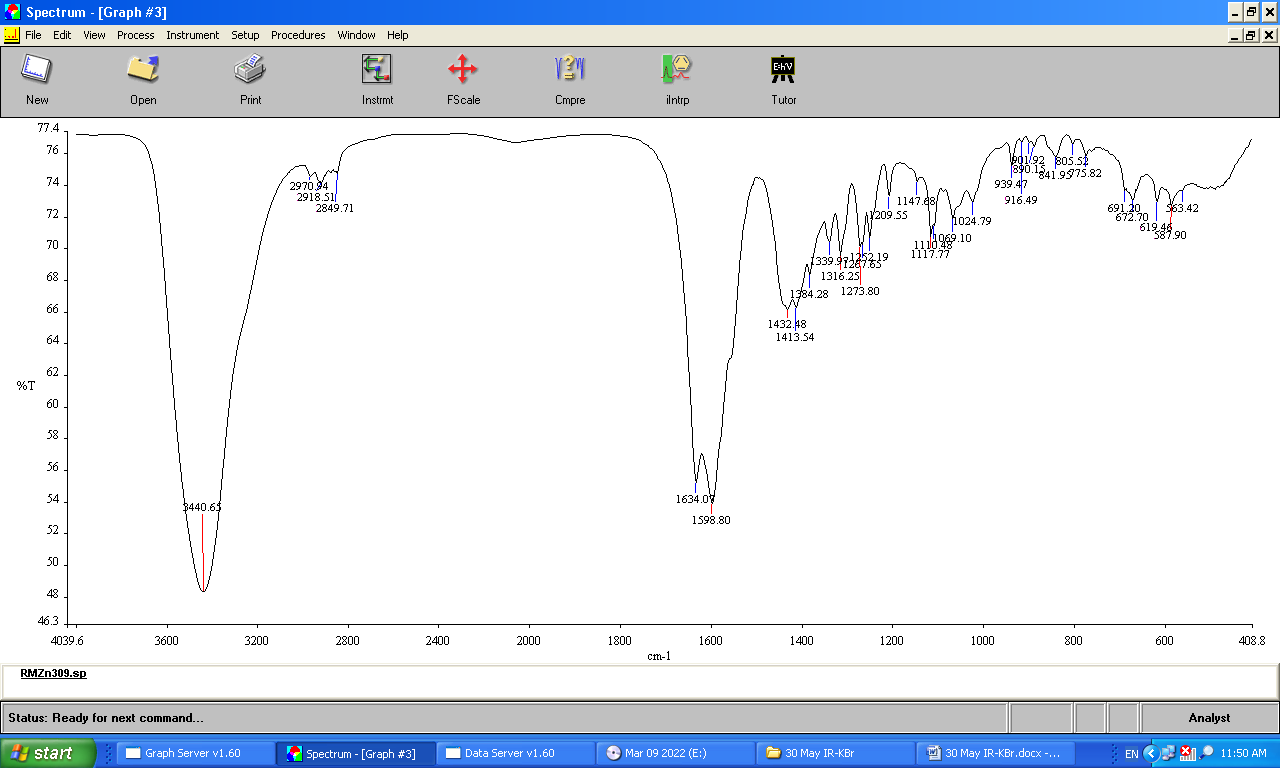


**Figure S6** IR spectrum of [Zn(L)(OAc)(H_2_O)].3H2O.0.5 EtOH


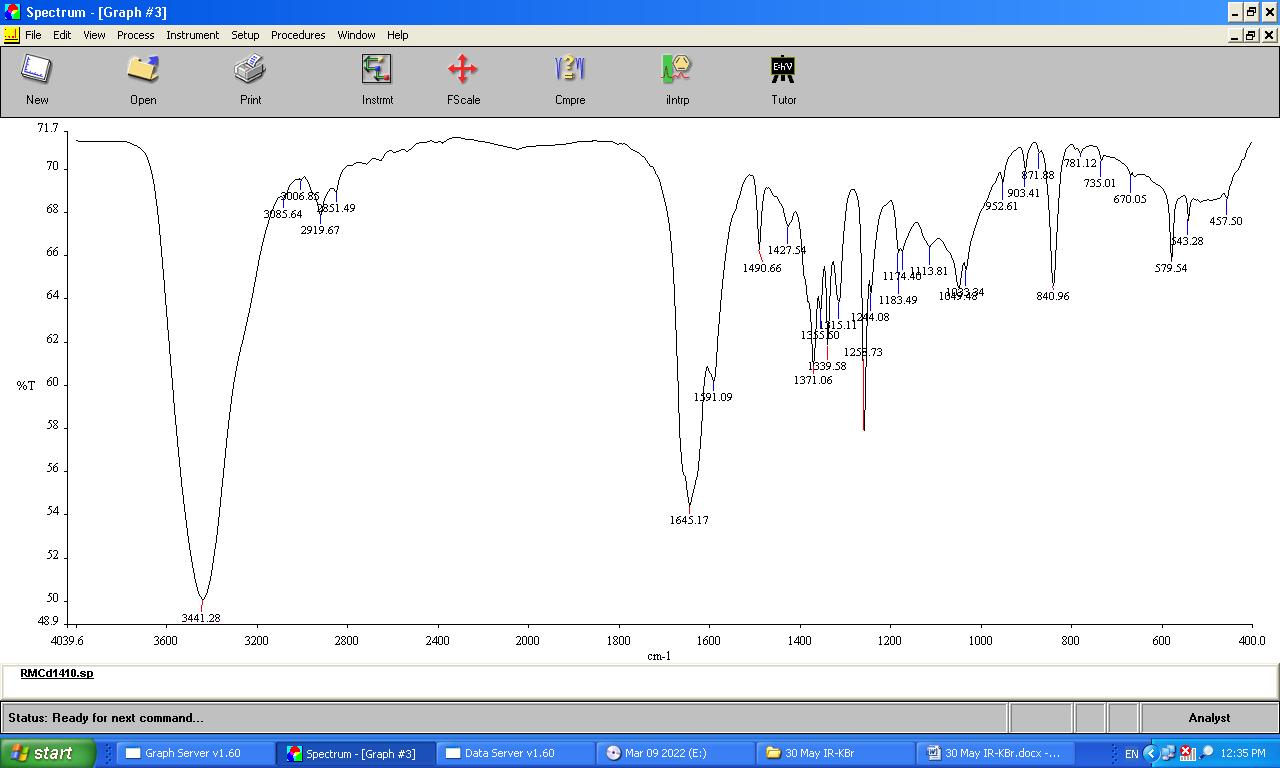


**Figure S7** IR spectrum of [Cd(L)(OAc)(H_2_O)].1.5H_2_O.2EtOH


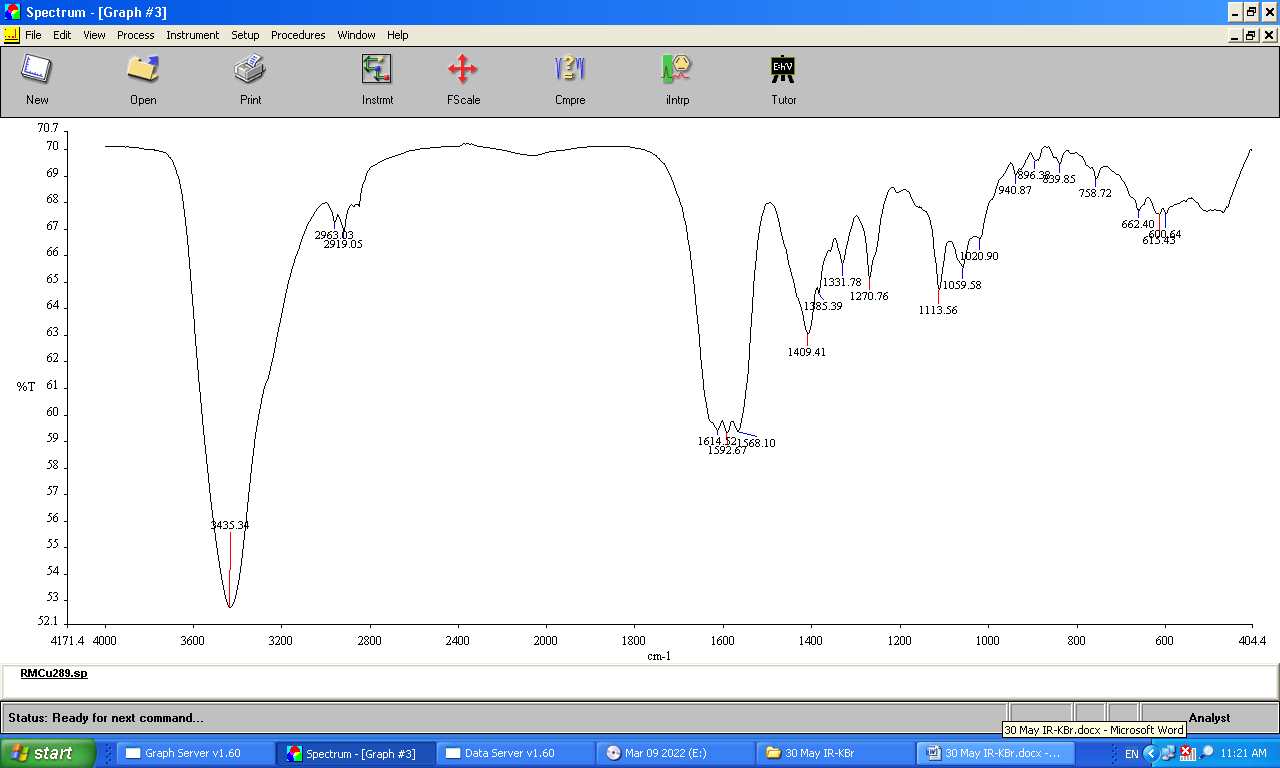


**Figure S8** IR spectrum of Cu(L)(OAc)(H_2_O)]


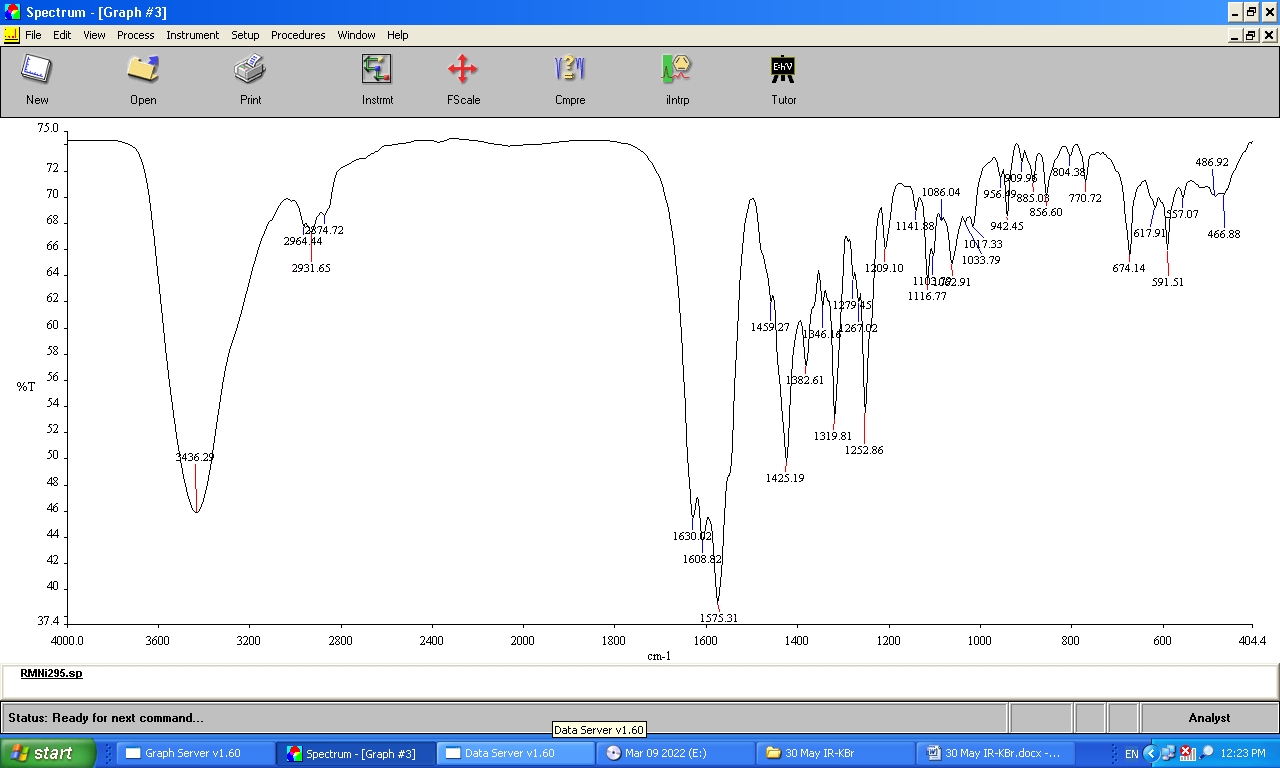


**Figure S9** IR spectrum of Ni(L)(OAc)(H_2_O)]


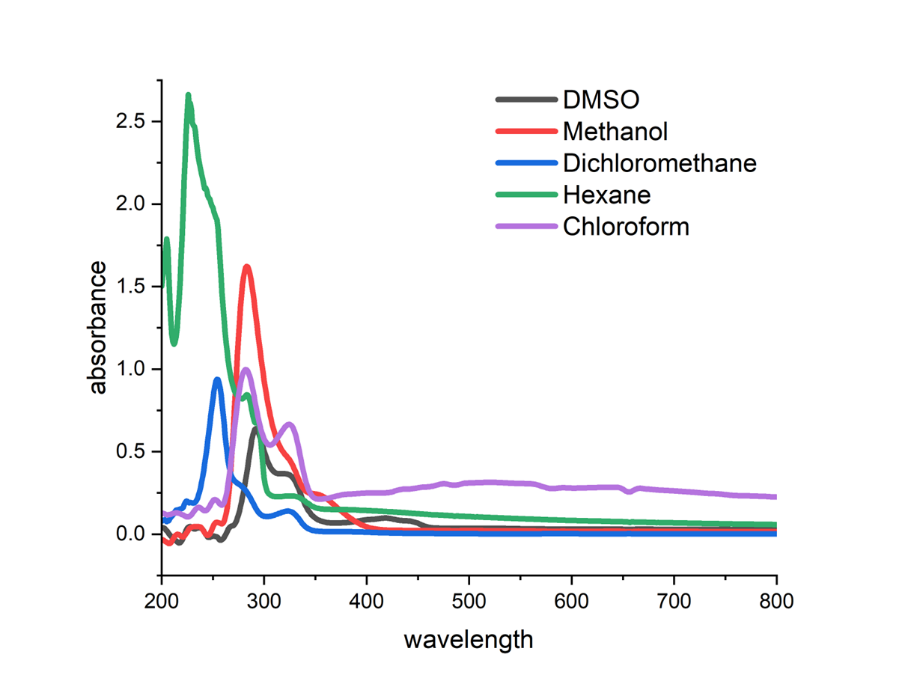


**Figure S10** Electronic UV–vis absorption of HL in nonpolar, and polar aprotic solvents.

**Figure S11.** Mass spectrum of HL.


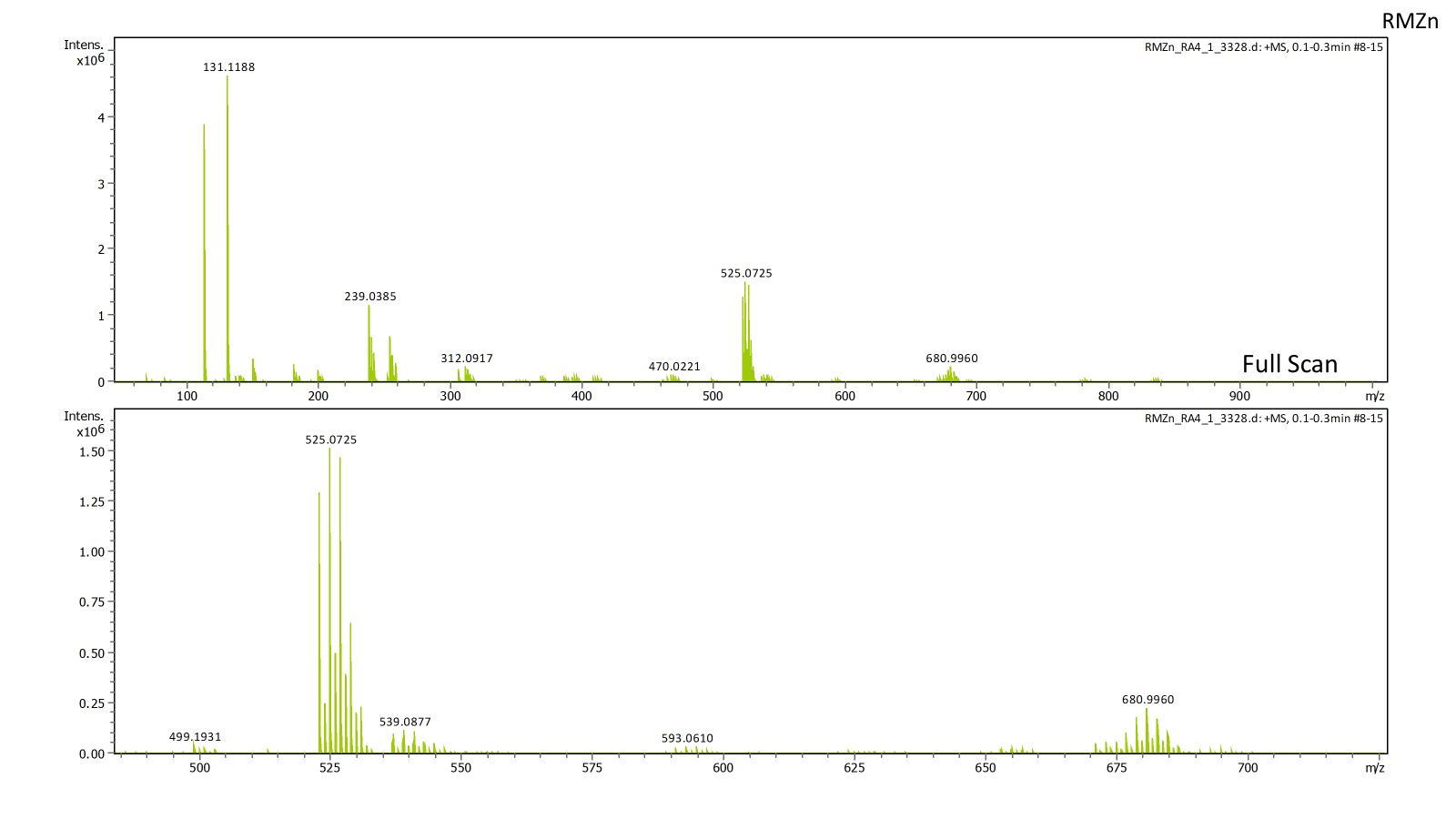


**Figure S12** Mass spectrum of [Zn(L)(OAc)(H_2_O)].3H_2_O.0.5 EtOH


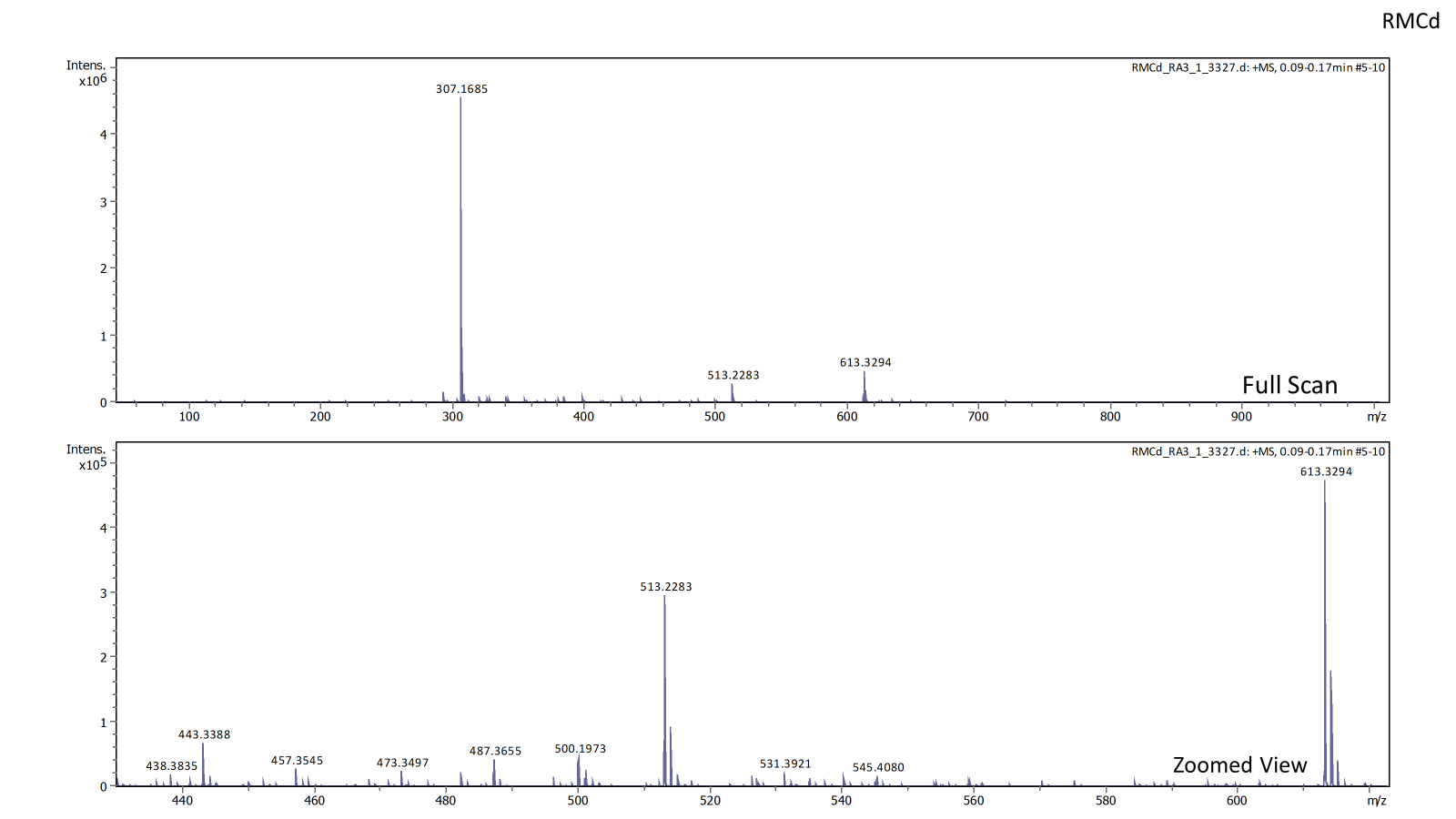


**Figure S13** Mass spectrum of [Cd(L)(OAc)(H_2_O)].1.5H_2_O.0.2EtOH


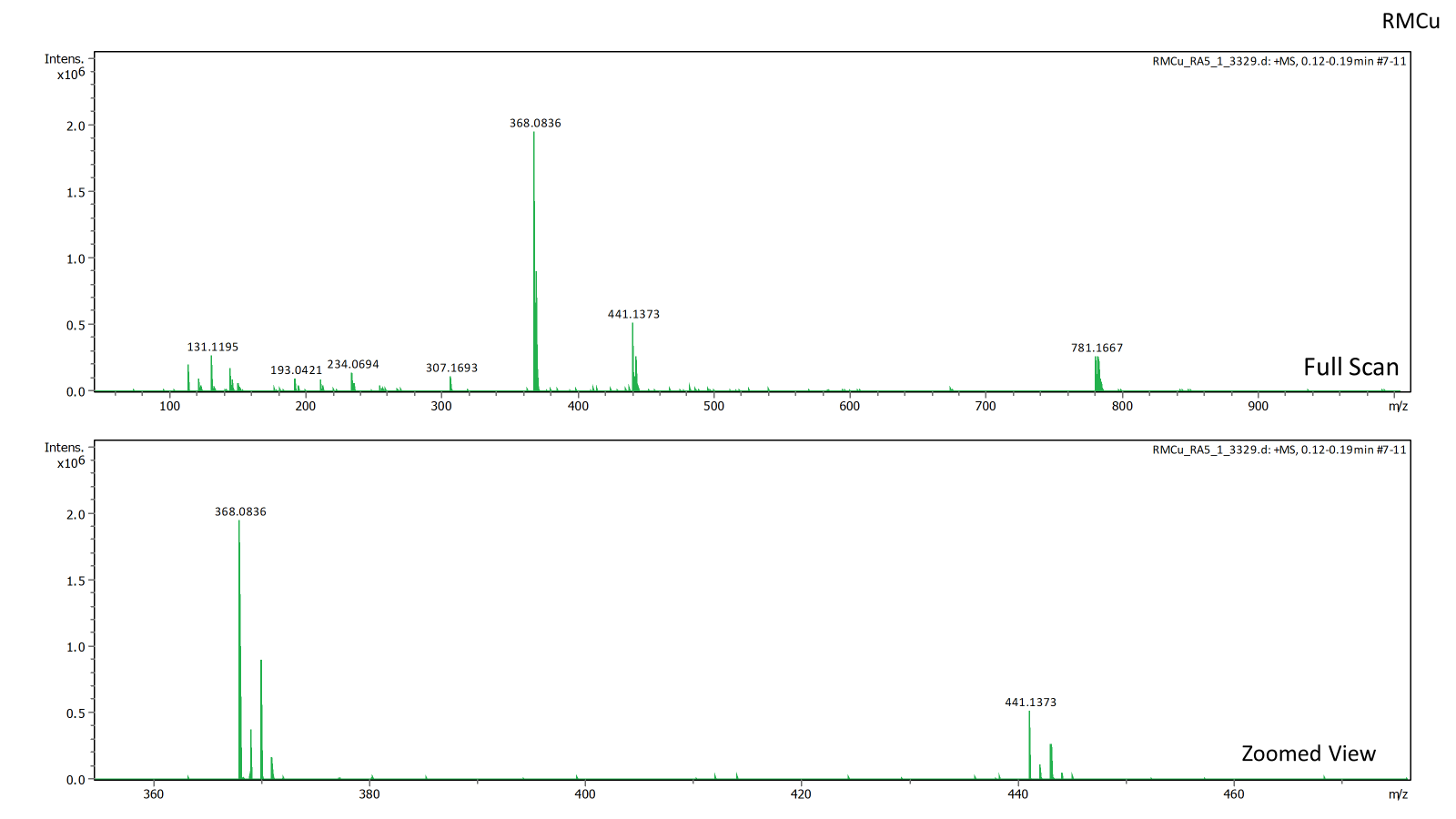


**Figure S14** Mass spectrum of [Cu(L)(OAc)(H_2_O)]


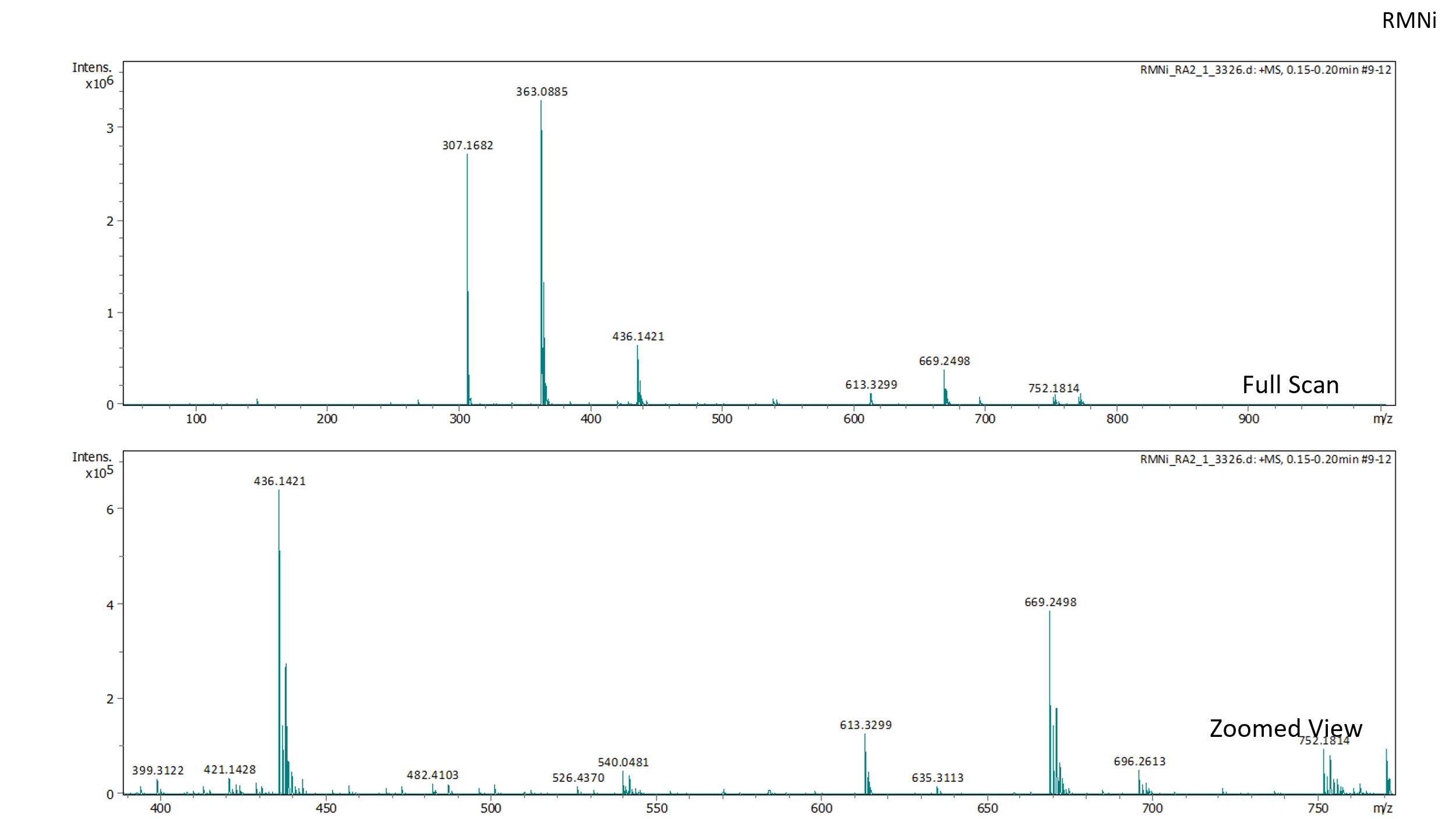


**Figure S15** Mass spectrum of [Ni(L)(OAc)(H_2_O)]


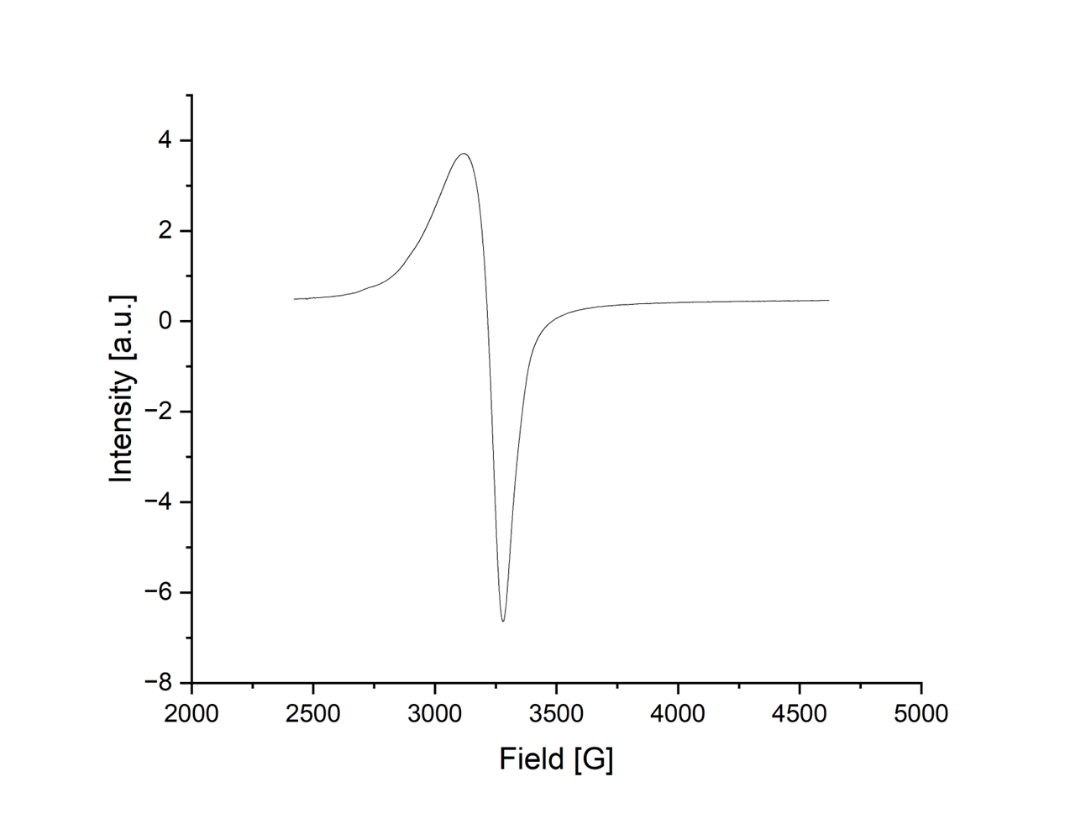


**Figure S16** ESR spectrum of Cu(II) Schiff base complex at 300 K.

| Comp. | 3D | 2D |
| --- | --- | --- |
| Ligand | 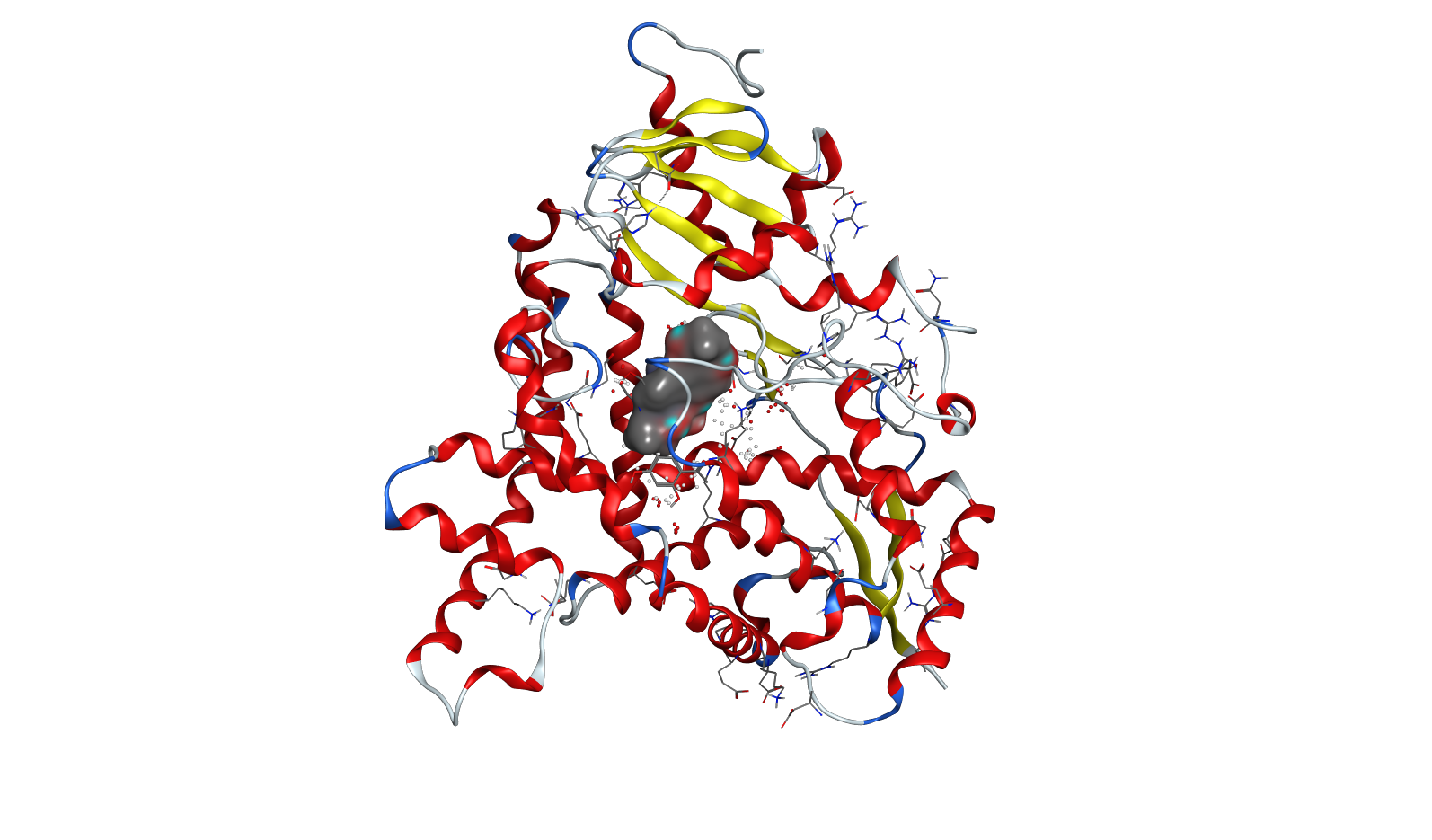 | 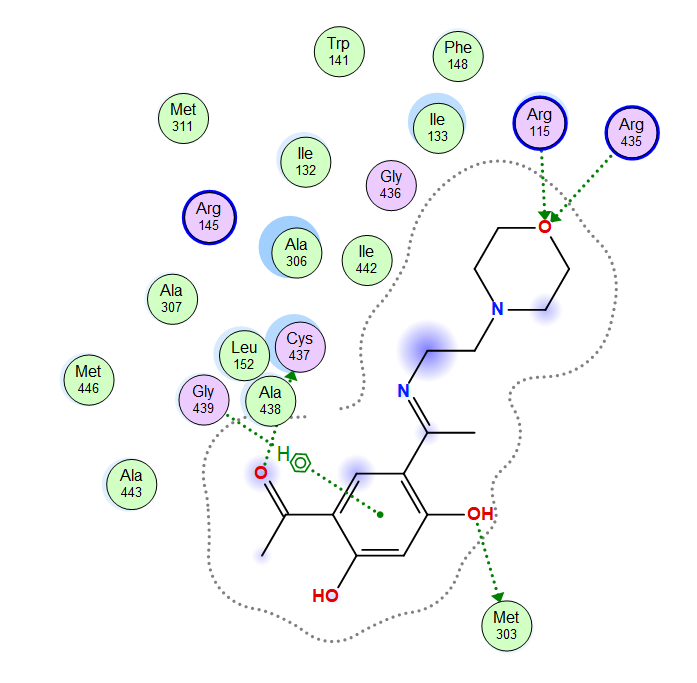 |
| Ni complex | 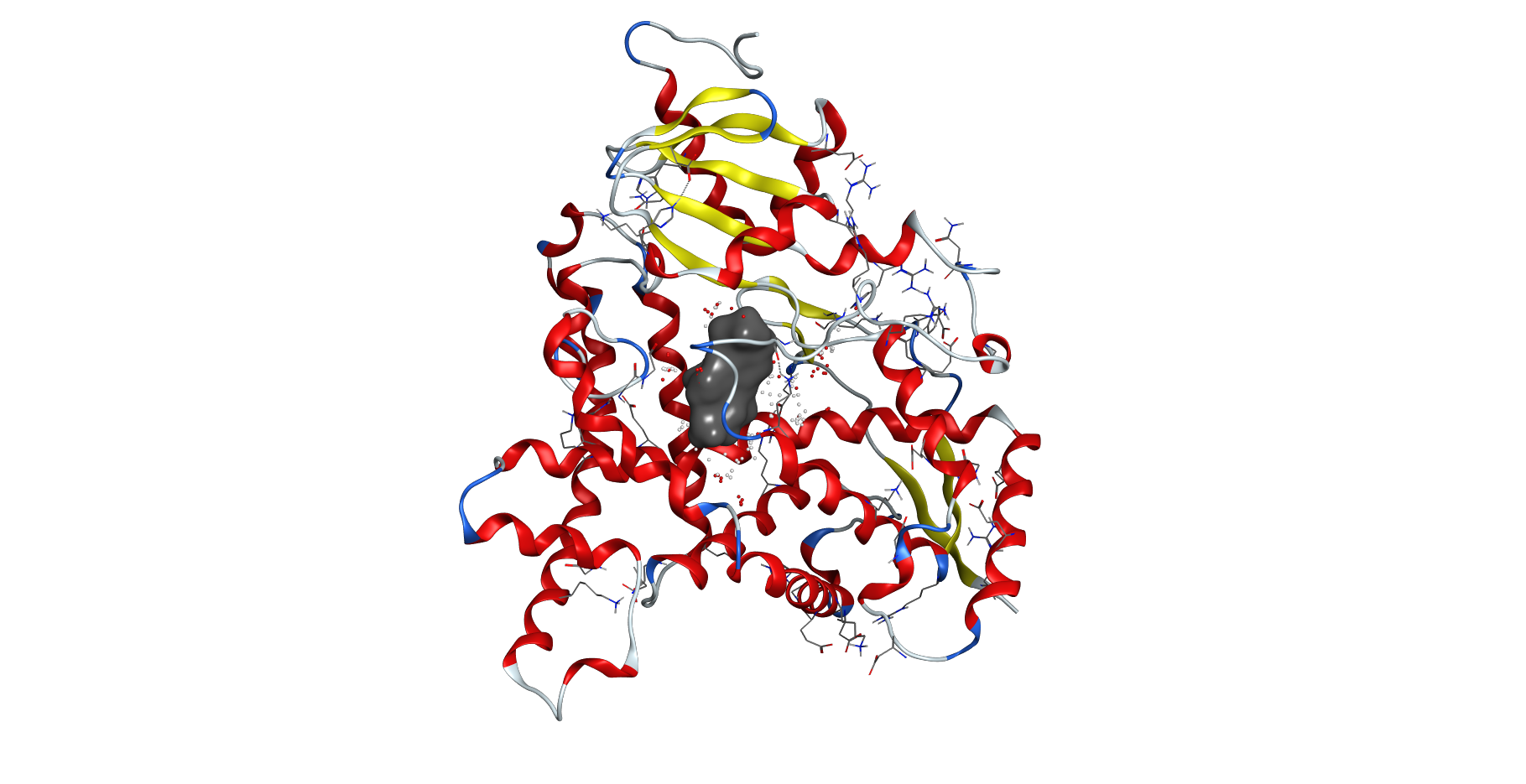 | 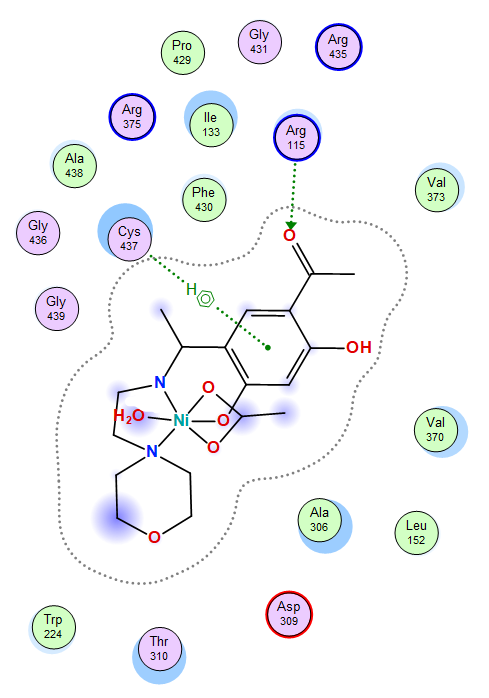 |
| Zn complex | 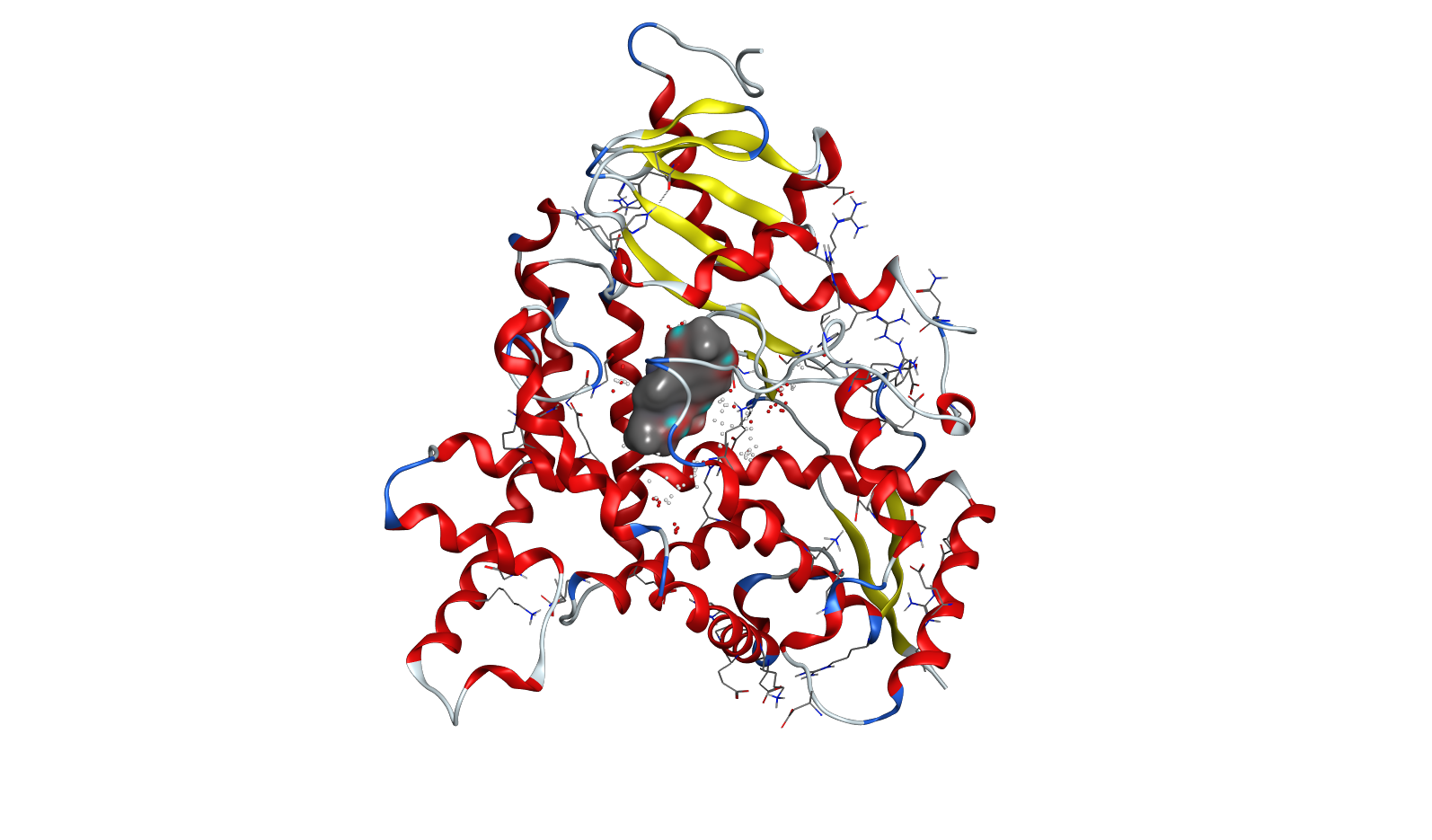 | 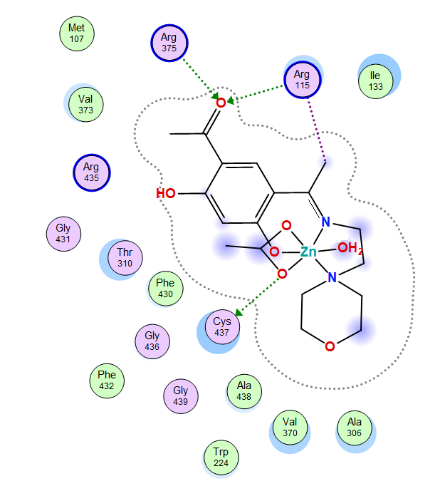 |
| Cd complex | 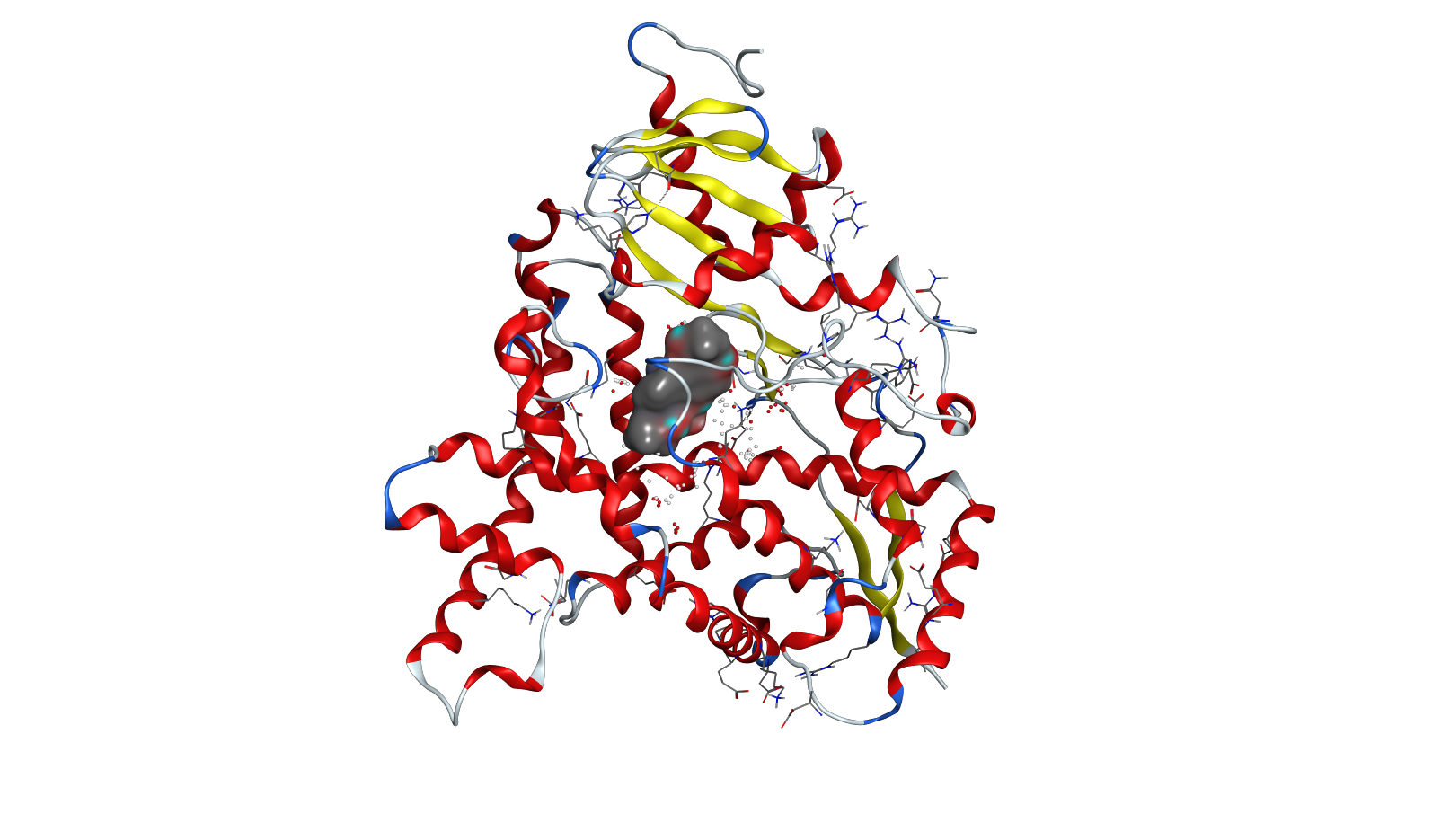 | 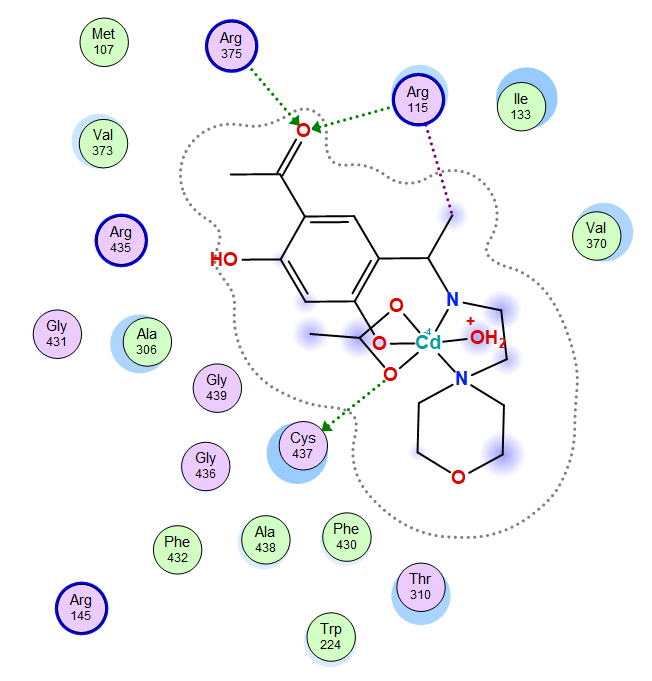 |

**Figure S17.** 2D, and 3D Docking interaction of the compounds and 3eqm as breast cancer protein

| **Crystal data** | |
| --- | --- |
| Chemical formula | C_16_H_22_N_2_O_4_ |
|  |  |
| *M*_r_ | 306.35 |
| Crystal system, space group | Monoclinic, *P*2_1_/*n* |
| Temperature (K) | 150 |
| *a*, *b*, *c* (Å) | 6.9300 (8), 22.531 (3), 9.9231 (11) |
| b (°) | 97.939 (5) |
| *V* (Å^3^) | 1534.6 (3) |
| *Z* | 4 |
| Radiation type | Cu *K*a |
| m (mm^-1^) | 0.79 |
| Crystal size (mm) | 0.44 × 0.04 × 0.02 |
| **Data collection** | |
| Diffractometer | Bruker AXS D8 Quest  diffractometer with PhotonIII_C14 charge-integrating and photon counting pixel array detector |
| Absorption correction | Multi-scan  *SADABS* 2016/2: Krause, L., Herbst-Irmer, R., Sheldrick G.M. & Stalke D., J. Appl. Cryst. 48 (2015) 3-10 |
| *T*_min_, *T*_max_ | 0.658, 0.754 |
| No. of measured, independent and  observed [*I* > 2s(*I*)] reflections | 17857, 3283, 2516 |
| *R*_int_ | 0.056 |
| (sin q/l)_max_ (Å^-1^) | 0.638 |
| **Refinement** | |
| *R*[*F*^2^ > 2s(*F*^2^)], *wR*(*F*^2^), *S* | 0.041, 0.111, 1.03 |
| No. of reflections | 3283 |
| No. of parameters | 209 |
| H-atom treatment | H atoms treated by a mixture of independent and constrained refinement |
| Dñ_max_, Dñ_min_ (e Å^-3^) | 0.22, -0.18 |

**Table S1.** Crystal Data and Structure Refinement of HL.

**Table S2.** Selected bond lengths (Å) and bond angles (°) for HL.

| **bond lengths (Å)** | | **bond lengths (Å)** | |
| --- | --- | --- | --- |
| O1—C4 | 1.2778 (17) | N1—C2—C3 | 117.99 (13) |
| O2—C6 | 1.3559 (17) | N1—C2—C1 | 119.28 (13) |
| N1—C2 | 1.3055 (18) | C3—C2—C1 | 122.74 (13) |
| C3—C8 | 1.394 (2) | C2—C3—C4 | 120.48 (13) |
| C3—C4 | 1.4606 (19) | O1—C4—C3 | 121.97 (13) |
| C2—C3 | 1.4462 (19) | O2—C6—C7 | 119.62 (13) |
| C1—C2 | 1.498 (2) | C2—N1—C11 | 126.54 (13) |
| O3—C9 | 1.2530 (17) | N1—C11—C12 | 109.11 (13) |
| C4—C5 | 1.422 (2) |  |  |
| C5—C6 | 1.367 (2) |  |  |
| C6—C7 | 1.4329 (19) |  |  |

| ***D*—H···*A*** | ***D*—H** | **H···*A*** | ***D*···*A*** | ***D*—H···*A*** |
| --- | --- | --- | --- | --- |
| O2—H2···O3 | 0.97(2) | 1.64(2) | 2.5369(17) | 151.9(19) |
| N1—H1···O1 | 0.931(18) | 1.700(18) | 2.5324(16) | 147.1(16) |
| C12—H12A···O3^i^ | 0.99 | 2.52 | 3.3852(19) | 146.1 |
| C16—H16A···O1^ii^ | 0.99 | 2.50 | 3.415(2) | 154.0 |
| Symmetry codes: (i) −*x*+1, −*y*+1, −*z*+1; (ii) *x*−1/2, −*y*+1/2, *z*+1/2. | | | | |

**Table S3.** Hydrogen bonds for HL (Å and °).

| **HL** | **Zn(II)** | **Cd(II)** | **Assignment** |
| --- | --- | --- | --- |
| 12.6 | 12.64 | 12.66 | (v.bs, 1H, -OH) |
| 8.19 | 8.07 | 8.27, 8.07 | (s, 1H, H_r-ring_) |
| 5.84 | 5.82 | 5.89, 5.81 | (s, 1H, H_r-ring_) |
| 3.76 | 3.71 | 3.70, 3.68 | (t, 2H, (-N-CH_2_) in ethylenic moiety) |
| 3.60 | 3.59 | 3.59, 3.55 | (t, 4H, -O-CH_2_) |
| 2.63 | 2.78 | 2.81, 2.77 | (t, 2H, (-CH_2_-N) in ethylenic moiety |
| 2.56 | 2.58 | 2.62, 2.56 | (t, 4H, (-N-CH_2_) in morpholine ring |
| 2.46 | ------------ | ----------------------------- | (bs, IH, CH_3_C-NH) |
| -------------- | 1.23 | 1.22 | (s, 3H, CH_3_COO) |
| 2.09 | 2.43, 1.81 | (2.53, 2.43) (1.83, 1.79) | (s, 6H, -CH_3_) |

**Table S4.** ^1^H NMR data of the ligand HL hand Zn(II) and Cd(II) complexes.

**Table S5.** Magnetic moments and electronic spectral data for complexes.

| **Ligand and complexes** | **λ_max_ (nm)** | **Band Assignment** | **µ_eff._ (B.M)** | **Geometry** | **d^n^** |
| --- | --- | --- | --- | --- | --- |
| HL | 300  325  425 | π-π*  π-π*  n-π* | - | - | - |
| [Zn(L)(OAc)(H_2_O)].3H_2_O.0.5 EtOH | 315  375 | π-π*  n-π* | Diamagnetic | Octahedral | d^10^ |
| [Cd(L)(OAc)(H_2_O)].1.5H_2_O.2 EtOH | 275  325 | π-π*  n-π* | Diamagnetic | Octahedral | d^10^ |
| [Cu(L)(OAc)(H_2_O)] | 300  350  600-800 | π-π*  n-π* | 1.77 | Octahedral | d^9^ |
| [Ni_2_(L)(OAc)(H_2_O)] | 325  360  625  770 | π-π*  n-π*  ^3^A_2g_(F)→^3^T_1g_(P)  ^3^A_2g_(F)→^3^T_1g_(F) | 3.12 | Octahedral | d^8^ |

n= number of electrons.

**Table S6.** Mass spectral data of the HL and the complexes.

| **Compound** | **Expected m/z** | **Found m/z** | **Peak assigned** |
| --- | --- | --- | --- |
| HL | 306.35 | 307.17 | [HL + H]^+^ |
| [Zn(L)(OAc)(H_2_O)].3H_2_O.0.5 EtOH | 524.88 | 525.07 | [M]^+^ |
| [Cd(L)(OAc)(H_2_O)].1.5H_2_O.0.2 EtOH | 613.98 | 613.33 | [M]^+^ |
| [Cu(L)(OAc)(H_2_O)] | 445.10 | 441.14 | [M + 4H]]^+^ |
| [Ni(L)(OAc)(H_2_O)] | 440.11 | 436.14 | [M + 4H]^+^ |

**Table S7.** Docking score and energy (kcal/mol) of the compounds and 3eqm as breast cancer protein

|  | S | rmsd_refine | E_conf | E_place | E_score1 | E_refine | E_score2 | E_orig |
| --- | --- | --- | --- | --- | --- | --- | --- | --- |
| Ligand | -6.49 | 1.17 | 63.66 | -80.00 | -10.37 | -32.80 | -6.49 | 0.60 |
|  | -6.31 | 1.42 | 55.63 | -75.90 | -10.76 | -36.70 | -6.31 | 0.60 |
|  | -6.18 | 1.02 | 57.69 | -82.10 | -10.41 | -29.04 | -6.18 | 0.00 |
|  | -6.06 | 2.14 | 63.89 | -106.09 | -11.18 | -35.86 | -6.06 | 2.82 |
|  | -6.03 | 2.83 | 61.67 | -77.63 | -10.09 | -41.34 | -6.03 | 2.40 |
| Ni complex | -7.44 | 1.97 | -712.55 | -75.31 | -10.91 | -37.31 | -7.44 | 0.60 |
|  | -7.22 | 1.88 | -712.93 | -70.04 | -12.01 | -34.32 | -7.22 | 0.60 |
|  | -7.01 | 3.22 | -711.24 | -70.26 | -11.14 | -36.82 | -7.01 | 0.60 |
|  | -6.97 | 1.28 | -706.70 | -100.08 | -12.08 | -31.75 | -6.97 | 0.60 |
|  | -6.76 | 2.50 | -710.54 | -91.71 | -10.79 | -32.60 | -6.76 | 0.00 |
| Cu complex | -8.13 | 1.95 | -604.08 | -75.31 | -10.91 | -28.51 | -8.13 | 0.60 |
|  | -7.55 | 1.78 | -608.99 | -70.04 | -12.01 | -23.04 | -7.55 | 0.60 |
|  | -7.50 | 2.20 | -605.90 | -68.40 | -10.59 | -20.80 | -7.50 | 0.60 |
|  | -6.27 | 3.98 | -604.56 | -90.14 | -10.72 | -22.41 | -6.27 | 0.60 |
|  | -6.14 | 2.76 | -605.87 | -91.71 | -10.73 | -22.70 | -6.14 | 0.00 |
| Zn complex | -7.80 | 2.03 | -518.97 | -75.31 | -10.91 | -33.32 | -7.80 | 0.60 |
|  | -7.67 | 1.33 | -514.52 | -100.08 | -12.08 | -24.41 | -7.67 | 0.60 |
|  | -6.65 | 1.64 | -523.38 | -70.04 | -12.01 | -24.59 | -6.65 | 0.60 |
|  | -6.64 | 1.43 | -506.98 | -82.71 | -11.36 | -18.79 | -6.64 | 0.60 |
|  | -6.57 | 4.06 | -519.53 | -95.26 | -10.94 | -31.94 | -6.57 | 0.60 |
| Cd complex | -7.55 | 1.99 | -471.81 | -75.31 | -10.91 | -33.07 | -7.55 | 0.60 |
|  | -7.11 | 4.14 | -471.53 | -90.14 | -10.72 | -34.97 | -7.11 | 0.60 |
|  | -6.84 | 1.40 | -465.90 | -100.08 | -12.08 | -25.00 | -6.84 | 0.60 |
|  | -6.66 | 1.70 | -476.19 | -70.04 | -12.01 | -23.69 | -6.66 | 0.60 |
|  | -6.56 | 4.26 | -484.73 | -95.26 | -10.94 | -28.85 | -6.56 | 0.60 |

**Table S8.** Docking interaction and energies of compounds and breast cancer protein

|  | Ligand | Receptor | Interaction | Distance | E (kcal/mol) |
| --- | --- | --- | --- | --- | --- |
| Ligand | O 15 | SG CYS 437 | H-donor | 3.46 | -2.1 |
|  | O 37 | SD MET 303 | H-donor | 3.13 | -3.0 |
|  | O 30 | NH2 ARG 115 | H-acceptor | 2.68 | -2.7 |
|  | O 30 | NE ARG 435 | H-acceptor | 3.03 | -3.3 |
|  | 6-ring | CA GLY 439 | pi-H | 4.34 | -0.5 |
| Ni complex | O 15 | NH1 ARG 115 | H-acceptor | 2.92 | -2.3 |
|  | O 15 | NH2 ARG 115 | H-acceptor | 2.78 | -2.7 |
|  | 6-ring | CB CYS 437 | pi-H | 3.68 | -0.5 |
| Cu complex | O 41 | SG CYS 437 | H-donor | 3.35 | -1.10 |
|  | O 15 | NH1 ARG 115 | H-acceptor | 2.6 | -3.02 |
|  | O 15 | NH2 ARG 115 | H-acceptor | 3.19 | -2.30 |
|  | O 15 | NE ARG 375 | H-acceptor | 3.08 | -2.00 |
|  | C 38 | NH2 ARG 115 | Ionic | 3.26 | -3.00 |
| Cd complex | O 41 | SG CYS 437 | H-donor | 3.35 | -1.1 |
|  | O 15 | NH1 ARG 115 | H-acceptor | 3.04 | -2.6 |
|  | O 15 | NH2 ARG 115 | H-acceptor | 3.24 | -2.0 |
|  | O 15 | NE ARG 375 | H-acceptor | 3.04 | -2.2 |
|  | C 38 | NH2 ARG 115 | Ionic | 3.37 | -2.4 |
| Zn Complex | O 41 | SG CYS 437 | H-donor | 3.20 | -1.0 |
|  | O 15 | NH1 ARG 115 | H-acceptor | 3.04 | -2.5 |
|  | O 15 | NH2 ARG 115 | H-acceptor | 3.21 | -2.2 |
|  | O 15 | NE ARG 375 | H-accepto | 3.07 | -2.0 |
|  | C 38 | NH2 ARG 115 | Ionic | 3.36 | -2.5 |
